# Cytoplasmic long noncoding RNAs are frequently bound to and degraded at ribosomes in human cells

**DOI:** 10.1261/rna.053561.115

**Published:** 2016-06

**Authors:** Joana Carlevaro-Fita, Anisa Rahim, Roderic Guigó, Leah A. Vardy, Rory Johnson

**Affiliations:** 1 Centre for Genomic Regulation (CRG), 08003 Barcelona, Spain; 2 Universitat Pompeu Fabra (UPF), 08003 Barcelona, Spain; 3 Institut Hospital del Mar d'Investigacions Mèdiques (IMIM), 08003 Barcelona, Spain; 4 A*STAR Institute of Medical Biology, Singapore 138648, Singapore; 5 School of Biological Sciences, Nanyang Technological University, 637551 Singapore

**Keywords:** long noncoding RNA, ribosome, translation, cytoplasm, transposable element, ribosome profiling, degradation

## Abstract

Recent footprinting studies have made the surprising observation that long noncoding RNAs (lncRNAs) physically interact with ribosomes. However, these findings remain controversial, and the overall proportion of cytoplasmic lncRNAs involved is unknown. Here we make a global, absolute estimate of the cytoplasmic and ribosome-associated population of stringently filtered lncRNAs in a human cell line using polysome profiling coupled to spike-in normalized microarray analysis. Fifty-four percent of expressed lncRNAs are detected in the cytoplasm. The majority of these (70%) have >50% of their cytoplasmic copies associated with polysomal fractions. These interactions are lost upon disruption of ribosomes by puromycin. Polysomal lncRNAs are distinguished by a number of 5′ mRNA-like features, including capping and 5′UTR length. On the other hand, nonpolysomal “free cytoplasmic” lncRNAs have more conserved promoters and a wider range of expression across cell types. Exons of polysomal lncRNAs are depleted of endogenous retroviral insertions, suggesting a role for repetitive elements in lncRNA localization. Finally, we show that blocking of ribosomal elongation results in stabilization of many associated lncRNAs. Together these findings suggest that the ribosome is the default destination for the majority of cytoplasmic long noncoding RNAs and may play a role in their degradation.

## INTRODUCTION

The past decade has witnessed the discovery of tens of thousands of long non-protein-coding RNAs (lncRNAs) in our genome, with profound implications for our understanding of molecular genetics, disease, and evolution. Focus is now shifting to understanding the function of these molecules, which is likely to be intimately linked to localization within the cell.

Following the first compelling discoveries of chromatin regulatory lncRNAs such as *XIST* (Brown et al. 1991) and *AIR* (Wutz et al. 1997; Lyle et al. 2000), a paradigm was established for lncRNAs as nuclear-restricted, epigenetic regulatory molecules (Khalil et al. 2009). However, it is not clear to what extent this is true for the >10,000 lncRNAs that remain uncharacterized (Cabili et al. 2011; Derrien et al. 2012; Hangauer et al. 2013; Managadze et al. 2013). Growing evidence points to lncRNAs having diverse roles outside of the cell nucleus, including regulation of microRNA activity (Cesana et al. 2011), protein sequestration (Kino et al. 2010), and mRNA translation (Carrieri et al. 2012).

Somewhat paradoxically, cytoplasmic lncRNAs have recently been reported to interact with the ribosome. In footprinting experiments to map ribosome-bound transcripts genome-wide, the Weissman group identified a considerable number of lncRNAs directly engaged by the translation machinery (Ingolia et al. 2011), an observation subsequently supported in an independent study (van Heesch et al. 2014). The functional relevance of these observations remains unclear, and the original proposal that lncRNAs are translated into functional peptides has not been supported by other studies (Banfai et al. 2012; Guttman et al. 2013). These transcripts do not contain classical features of protein-coding sequence and various analyses have argued that they are not productively translated in most cases (Banfai et al. 2012; Chew et al. 2013; Guttman et al. 2013). Furthermore, it is likely that early footprinting experiments suffered from a significant false-positive rate in ribosome-binding predictions (Ingolia et al. 2014). Unfortunately, while sensitive, these techniques do not allow absolute estimates of the cellular pool of lncRNA molecules involved in ribosomal interactions. Hence, the biological significance of this phenomenon has not been established.

Here we address this question by mapping a stringently filtered lncRNA population within the cytoplasm and polysomes of a human cell line. We estimate the relative ribosome-associated and free populations of lncRNA, which are verified by quantitative PCR and validated by puromycin-mediated disruption of ribosomes. We show evidence that lncRNAs can be divided into classes based on ribosomal association, and these classes are distinguished by a variety of features, most notably transposable element insertions and mRNA-like features at the 5′ end. Finally, we show that these lncRNAs are sensitive to drug-induced stalling of ribosomes, implicating degradation as one outcome of lncRNA-ribosome interactions.

## RESULTS

### Mapping the cytoplasmic and ribosome-associated lncRNA population

**FIGURE 1.**
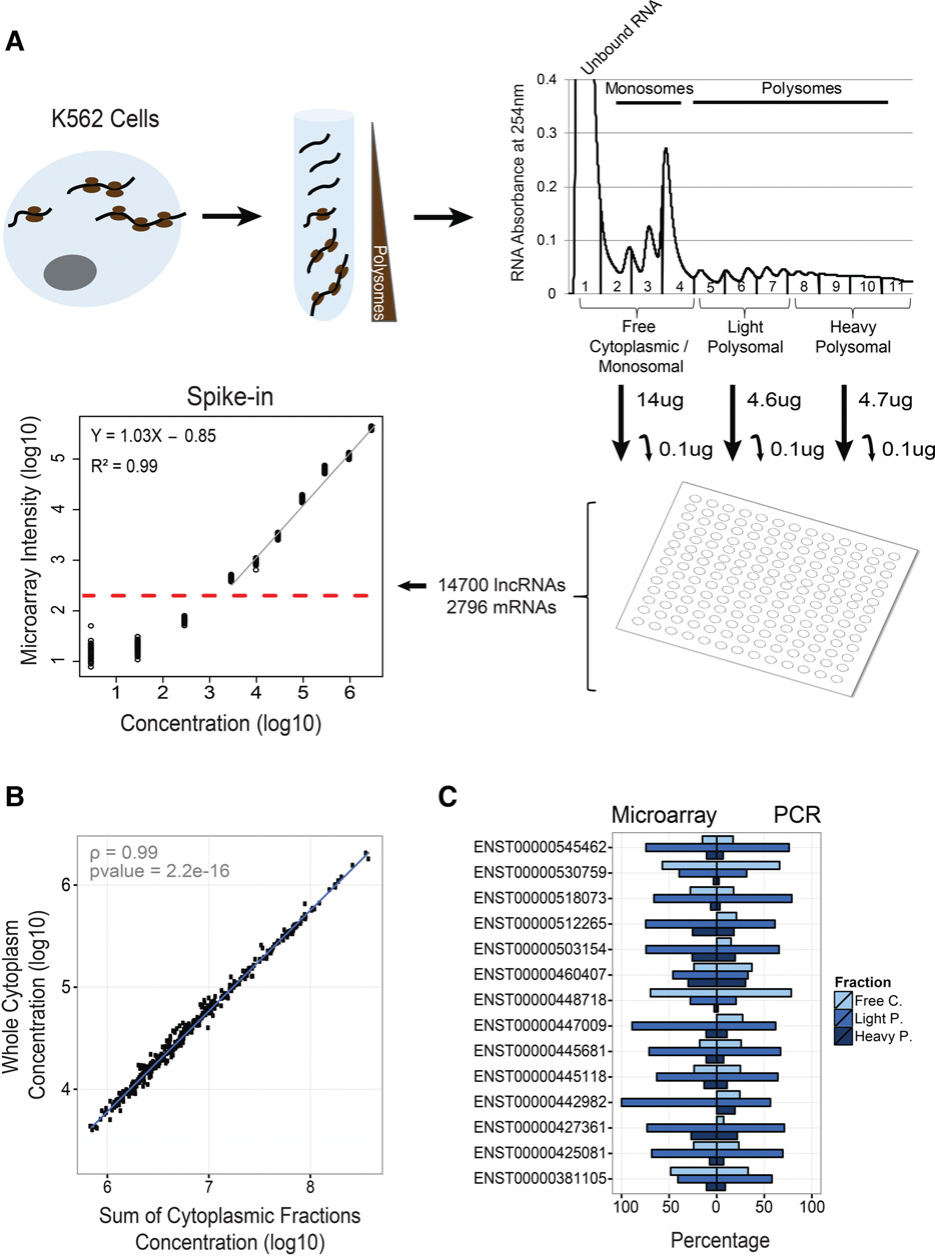
Discovery and quantification of ribosome-associated lncRNAs by polysome profiling and microarray hybridization. (*A*) Outline of the subcellular mapping of K562 lncRNA by polysome profiling and microarray hybridization. Sucrose-gradient ultracentrifugation was used to isolate the indicated fractions of ribosome-associated RNA, quantifications of which are displayed at the *upper right*. The pooled fractions used in this study are shown *below* the figure. The total amount of RNA isolated from each fraction is indicated by arrows, from which 0.1 µg was collected and hybridized to custom lncRNA microarrays. Microarrays were normalized using spike-ins: At the *lower left* is shown a representative example of the linear regression of spike in probe intensity against their starting concentrations. Dashed red line represents the defined detection threshold for this fraction where regression ceases to be linear. Only probes above this threshold were considered detected. (*B*) Correlation of the sum of the three cytoplasmic fraction concentration estimates and total cytoplasmic concentration estimate, supporting the quantification approach used. (*C*) Barplot shows for 14 lncRNA examples the relative amount (expressed as a percentage) of transcript molecules estimated to be present in each of the fractions. Sum of percentages of the three fractions has to be 100%, the total of detected molecules in the cytoplasm. *Left* bars represent quantification by microarrays and *right* bars by the mean of two quantitative PCR biological replicates. Microarray and PCR experiments represent different biological replicates.

We sought to create a comprehensive and quantitative map of cytopasmic lncRNA localization in a human cell. We chose as a model the K562 human myelogenous leukemia cell line because, as an ENCODE Tier I cell, it has extensive transcriptomic, proteomic, and epigenomic data publicly available (Djebali et al. 2012). We subjected cytoplasmic cellular extracts to polysome profiling, an ultracentrifugation method to identify ribosome-bound RNAs and distinguish transcripts bound to single or multiple ribosomes (Rahim and Vardy 2016). Consistent with previous studies (Zhang et al. 2012; Wong et al. 2016), extracts were divided into three pools: “heavy polysomal,” corresponding to high molecular weight complexes cofractioning with greater than six ribosomes; “light polysomal,” cofractioning with two to six ribosomes; and low-molecular weight complexes corresponding to nontranslated, cytoplasmic RNAs (Fig. 1A). The latter contains free mRNAs found in the high peak in fraction 1, the 40 and 60S ribosomal subunits (fractions 2 and 3) and mRNAs that are bound by a single ribosome (fraction 4)—we define these as “free cytoplasmic” throughout the paper. It is important to note that although this fraction includes some RNAs bound by ribosomal subunits, or individual ribosomes, the majority of these are not considered to be efficiently translated (Ruan et al. 1997; Zhang et al. 2012).

We designed a microarray hybridization experiment to estimate the relative amounts of cytoplasmic lncRNA present in the three fractions above (see Materials and Methods for details). Custom microarrays probing the entire Gencode v7 long noncoding RNA catalog were used to profile RNAs in the free cytoplasmic, light and heavy polysomal fractions, in addition to the total input RNA (Derrien et al. 2012). Microarrays also contained probes targeting 2796 protein-coding genes. Data were normalized absolutely to spiked-in synthetic external RNA added to samples at known concentrations prior to hybridization, in order to control for unwanted sources of variability (Fig. 1A), and then further normalized by the relative starting amounts of RNA purified from the same cell lysate. In this way we estimated the relative cellular concentrations of free and polysomal concentrations of all lncRNAs in K562 cells.

We used two buffer conditions with differing stringency to extract the polysomal-associated RNAs (Materials and Methods). We observed essentially no difference in array quantifications between high and low stringency buffer conditions: The estimated concentrations for protein-coding mRNAs (Supplemental Fig. S1) and lncRNAs (Supplemental Fig. S2) were unaffected by increasing buffer stringency, with Pearson correlation coefficients of 0.99, 0.99, and 0.99 (*P* < 2.2 × 10^−16^, Pearson correlation test) between buffer conditions for heavy polysomal, light polysomal, and free cytoplasmic transcripts, respectively. Thus, we proceeded to perform further analysis using only microarray quantifications from higher stringency buffer conditions.

Further supporting the validity of this approach, we observed close correlation (cor = 0.99, *P* = 2.2 × 10^−16^) between the sum of the estimated concentrations across fractions, and that of a separate hybridization of total cytoplasmic RNA from the same cells (Fig. 1B). Quantitative PCR carried out on the same samples also supported the microarray estimation (cor = 0.89, *P* = 1.5 × 10^−15^) (Fig. 1C).

### Creating a high confidence lncRNA catalog

**FIGURE 2.**
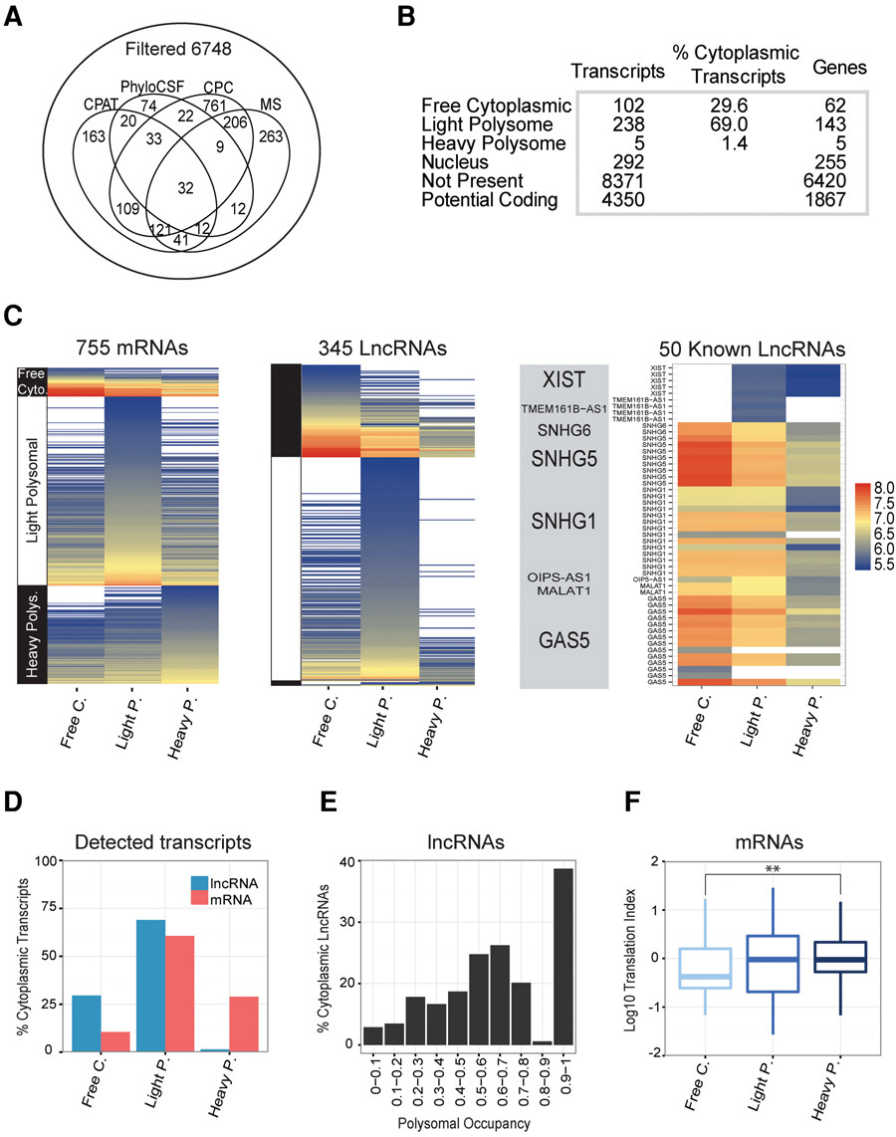
Classification and measurement of lncRNAs across cytoplasmic and ribosomal fractions. (*A*) Creating a high confidence non-protein-coding Gencode v7 lncRNA gene set. Genes (and all their constituent transcripts) having at least one transcript identified as protein coding by at least one method were designated “potential protein-coding.” Remaining genes with no evidence for protein-coding potential were defined as “filtered lncRNAs.” (*B*) Summary of the numbers of genes and transcripts classified by polysome association. (*C*) Heatmaps showing lncRNAs log_10_ concentration measured for each RNA fraction. Only lncRNA transcripts and protein-coding genes detected in at least one cytoplasmic fraction are shown. “Known lncRNAs” are those filtered transcripts that also belong to the lncRNAdb database (Amaral et al. 2011). (*D*) Barplot shows the percentage of cytoplasmic lncRNAs and protein-coding genes classified in each cytoplasmic fraction. Transcripts and genes are classified in the fraction where they display maximal detection. (*E*) Barplot shows the percentage of cytoplasmic lncRNAs classified in each polysomal occupancy value bin. Polysomal occupancy value represents the ratio of polysomal (the sum of Light and Heavy fractions) to total cytoplasmic RNA. (*F*) Boxplot shows translation index for mRNAs classified as free cytoplasmic, light polysomal, and heavy polysomal. Translation index is defined as the log_10_ ratio of mRNA-associated peptides expression to mRNA level, assayed in K562 by mass spectrometry and microarray, respectively. Statistical significance was calculated by Kolmogorov–Smirnov test ([**] *P* = 0.01).

A potential confounding factor in any analysis of ribosome-bound RNAs is the possibility of misannotated protein-coding transcripts of various types (Dinger et al. 2008; Kondo et al. 2010; Slavoff et al. 2013). We implemented a stringent filtering step to remove protein-coding transcripts from our analysis, even at the expense of omitting some genuine noncoding transcripts. We first removed lncRNAs that could be unannotated extensions of protein-coding genes or pseudogenes. The remaining genes were filtered using a panel of computational methods for identifying protein-coding sequence (Fig. 2A and Materials and Methods). Altogether 9008 lncRNA transcripts (62.1%) (6748 genes, 73.9% of total) were unanimously classified as noncoding—these we refer to as “filtered lncRNAs” (Fig. 2A). The remaining genes of uncertain protein-coding status are henceforth referred to as “potential protein-coding RNAs” (4350 transcripts, 1867 genes). The complete sets of potential protein-coding and filtered lncRNAs are available in Supplemental Table S1.

Using stringent cutoffs (see Materials and Methods) we detected 3.8% of filtered lncRNA transcripts (345 transcripts, representing 205 or 3.2% of genes) and 27% of mRNAs (755) in K562 cytoplasm. From the remaining filtered lncRNA transcripts, an additional 292 transcripts (3.2%, representing 255 or 3.7% genes) were detected in the nucleus based on ENCODE data (see Materials and Methods), and henceforth defined as nuclear-specific. Consistent with this, and as shown below, these transcripts are enriched in the nuclear compartment of multiple ENCODE cell lines. Altogether, 637 filtered lncRNA transcripts (7%, representing 460 or 6.8% of genes) were detected in K562 (Fig. 2B).

We classified cytoplasmic lncRNAs according to their maximal ribosomal association, resulting in 102 (29.6% of cytoplasmic lncRNA transcripts) free cytoplasmic, 238 (69%) light polysomal, and five (1.4%) heavy polysomal transcripts (Fig. 2B,C). Altogether, 70.4% of lncRNA transcripts detected in the cytoplasm have maximal detection in light or heavy polysomal fractions, hereafter collectively referred to as “polysomal” transcripts (Fig. 2D). These findings are essentially identical in a replicate experiment with a lower stringency buffer: 92.5% of the transcripts are consistently classified (Supplemental Fig. S3). Defining polysomal occupancy as the ratio of polysomal to total cytoplasmic RNA, we find lncRNAs spanning the entire range, peaking between 50% and 60% (Fig. 2E). Almost one-quarter of lncRNA examined have >90% of signal detected in polysomal fractions.

Two lines of evidence support this classification approach. First, 29% (218/755) of protein-coding mRNAs are classified as heavy polysomal, consistent with their being actively translated and in accordance with previous studies (Fig. 2C; Beilharz and Preiss 2004; Zhang et al. 2012). Second, protein abundance measurements show that polysomal mRNAs are translated most efficiently. Both light and heavy polysomal mRNAs have a higher translation index (defined as the ratio of peptide expression to mRNA concentration) compared to free cytoplasmic mRNAs (Fig. 2F). Translation index positively correlates with polysomal occupancy of mRNAs (Pearson correlation: cor = 0.03 *P* = 0.6; cor = 0.16 *P* = 0.02; cor = 0.2 *P* = 0.006 for translation index versus free cytoplasmic, light, and heavy polysomal occupancy, respectively; Supplemental Fig. S4). Thus, we observe the expected trend for mRNAs to be more efficiently translated in heavy polysomal fractions. The relatively low correlation observed between translation index and polysome association may be a result of differences in the two methodologies (mass spectrometry and polysome profiling), the production of data in cell lines cultivated in different laboratories, and the importance of regulatory differences in translational rates between mRNAs that has been previously reported (Schwanhäusser et al. 2011).

In contrast, potential protein-coding transcripts had a similar global ribosome-association profile to filtered lncRNA, suggesting that they are not translated efficiently, and underlining the stringency of our lncRNA filtering (Supplemental Fig. S5).

Cytoplasmic and ribosomal localization has previously been reported for a number of lncRNA. To test the degree of agreement between these and our data, we examined the 297 lncRNA transcripts (from 60 genes) from the LncRNA Database (Amaral et al. 2011) that are also present in the Gencode v7 annotation. *SNHG5* (Derrien et al. 2012) and *Gas5* (Kino et al. 2010) were detected in the cytoplasm and classified as free cytoplasmic, consistent with previous reports. The snoRNA host *Gas5* has previously been reported as associated with ribosomes (Smith and Steitz 1998). Although we classified this gene as free cytoplasmic based on its maximal detection, 11 out of 14 transcript isoforms of *Gas5* were also clearly detected in light and heavy polysomal fractions, although with lower intensities. Another example is *SNHG6* gene, which although mostly localized in the cytoplasm, can also associate with ribosomes (Makarova and Kramerov 2005). Consistent with this, we classify this gene as free cytoplasmic, but with a substantial polysomal component. *SNHG1* is another snoRNA host reported to be bound by ribosomes (Pelczar and Filipowicz 1998), for which we classify eight out of 14 cytoplasmic isoforms in the light polysomal fraction. Interestingly, the widely studied noncoding RNA *MALAT1*, classified as free cytoplasmic in this study based on its maximum detection, is also detected in the polysomal fractions, consistent with previous ribosome footprinting studies (Ingolia et al. 2011; Wilusz et al. 2012) and the discovery of a peptide mapping to it (Gascoigne et al. 2012). *MALAT1* is also detected in K562 cytoplasmic extracts by RNA-seq, and in public ribosome footprinting data (Supplemental Fig. S6).

For other known lncRNAs, we map their subcellular localization for the first time, like *TMEM161B-AS1*, which is specifically associated with the light polysomal fraction. Most intriguingly, there is a weak but detectable signal for nuclear lncRNA *XIST* in light polysomes and we validated this interaction by puromycin treatment (Supplemental Fig. S7) (see below). This is consistent with previous reports that during cell division, *XIST* is released from the nucleus (Hall et al. 2009). Indeed, similar to *MALAT1*, *XIST* is also detected in ribosome footprinting experiments (Supplemental Fig. S6).

### Independent evidence for ribosomal interaction of lncRNA

**FIGURE 3.**
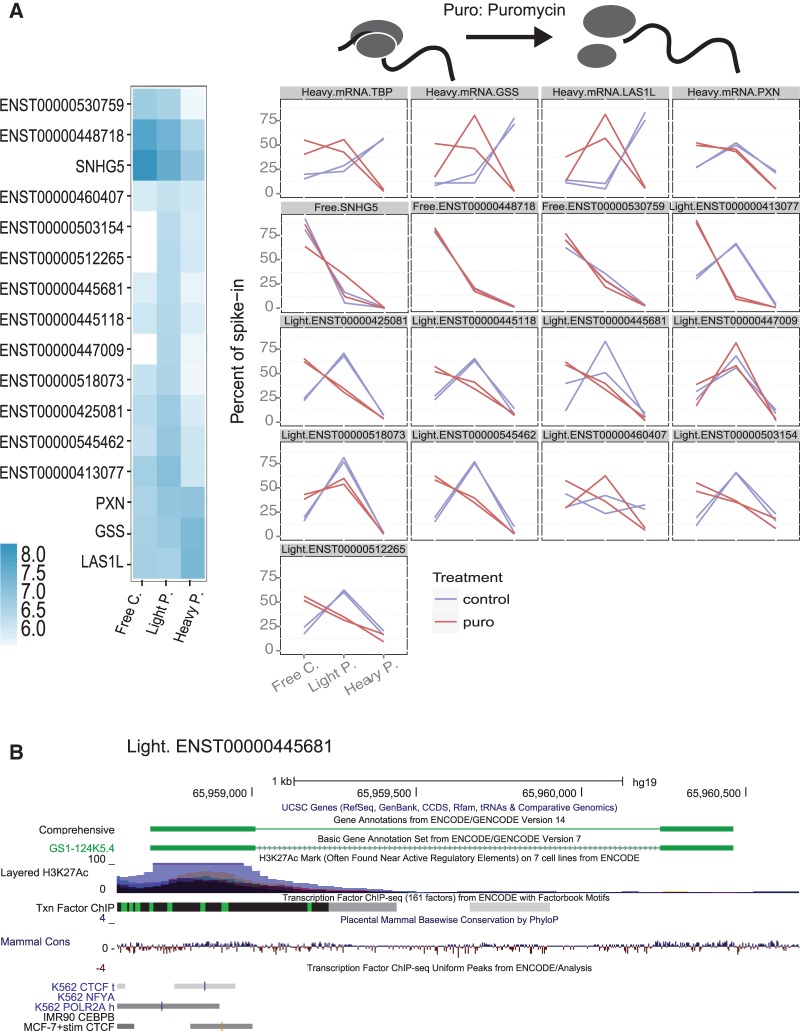
Validation of selected ribosome-associated lncRNA candidates. (*A*) qRT-PCR validation of ribosome-associated lncRNAs and free cytoplasmic lncRNAs in independent polysome profile experiments. In each case, two replicate experiments were carried out with control K562 cells (blue) and cells treated with puromycin (red) for three distinct RNA fractions. RNA levels are normalized to absolute levels of an RNA spiked into equal volumes of RNA sample. The *top* four panels represent protein-coding mRNAs. Transcript IDs and classifications are shown *above* each panel. The heatmap displays the log_10_ concentration values for the same genes predicted from the microarray. (*B*) Genomic map of ENST00000445681, an example of a ribosome-associated transcript validated above with evidence of evolutionary conservation and regulated transcription.

We next looked for additional evidence to support ribosomal interaction of lncRNAs. During ultracentrifugation, it is possible that lncRNAs associated with nonribosomal, high molecular weight complexes may co-sediment with polyribosomes and thus represent false positives. To investigate this, we repeated polysome profiling on cells treated with puromycin (puro), an aminoacyl-tRNA analog that selectively disrupts polysomes by causing premature chain termination, and profiled a set of candidate transcripts by volume-normalized RT-PCR (Fig. 3A). Bona fide ribosome-bound transcripts are expected to relocalize to the lighter polysome or free cytoplasmic fractions in response to puromycin. Nine out of 10 ribosome-associated lncRNAs were validated in this way, similar to the 4/4 protein-coding mRNAs tested. As expected, the free cytoplasmic lncRNAs were unaffected by the puromycin treatment. An example of a light polysomal lncRNA, ENST00000445681, is shown in Figure 3B. Thus in the majority of cases, co-sedimentation in polysome profiling reflects a genuine physical interaction between lncRNA and intact ribosomes.

**FIGURE 4.**
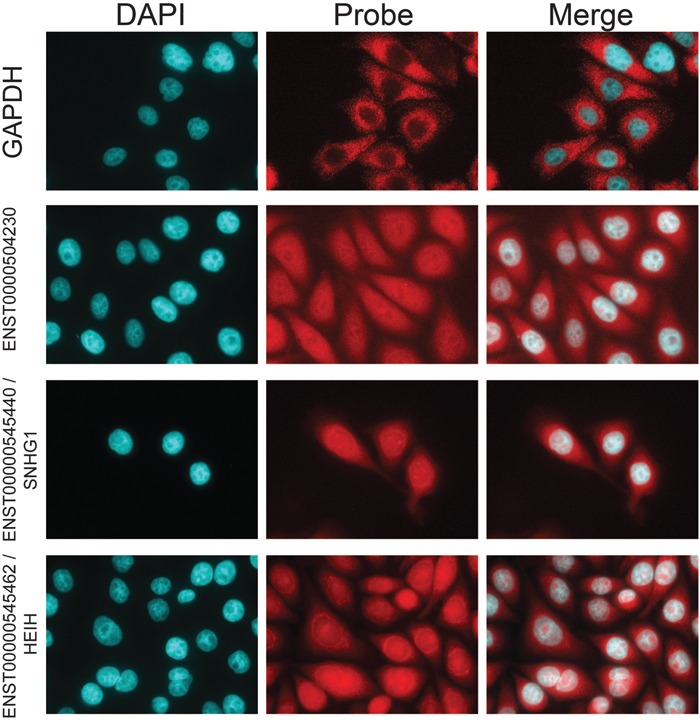
Fluorescence in situ hybridization of ribosome-associated lncRNAs in HeLa cells. (*Left* panel) DAPI staining of DNA; (*middle*) FISH probe; (*right*) merged. The actively translated housekeeping mRNA *GAPDH* was tested as a positive control for cytoplasmic localization.

We performed additional validation using fluorescence in situ hybridization (FISH) to visualize the localization of lncRNA at subcellular resolution. K562 cells grow in suspension, making FISH experiments challenging. Thus we performed FISH experiments in adherent HeLa cells, and tested three lncRNAs that are expressed and cytoplasmically localized in both cell lines (Fig. 4). ENST0000504230 displays diffuse cytoplasmic and nuclear localization with exclusion from nucleoli. In addition to cytoplasmic localization, the snoRNA precursor transcript ENST00000545440 (*SNHG1*) shows pronounced concentrations around the periphery of the nucleus, likely to be endoplasmic reticulum (ER), and at three nuclear loci—possibly its site of transcription, given that the HeLa genome is predominantly triploid (Adey et al. 2013). Finally, ENST00000545462 (also described as *HEIH*, a prognostic factor in hepatocellular carcinoma) (Yang et al. 2011), has pronounced staining in the nuclear periphery, as well as within the nucleolus and diffuse staining in the cytoplasm. Localization of these lncRNAs to the cytoplasm and possibly the ER supports their localization on translating polysomes. Thus, both PCR and hybridization methods support the interpretation from microarray data of ribosomal recruitment of lncRNA.

### Ribosome-associated lncRNAs are more homogeneously expressed and consistently localized across cell types

To gain insights into differences between ribosome-associated and free cytoplasmic lncRNAs, we next investigated whether these two groups of transcripts have different expression profiles across cell types and human tissues.

**FIGURE 5.**
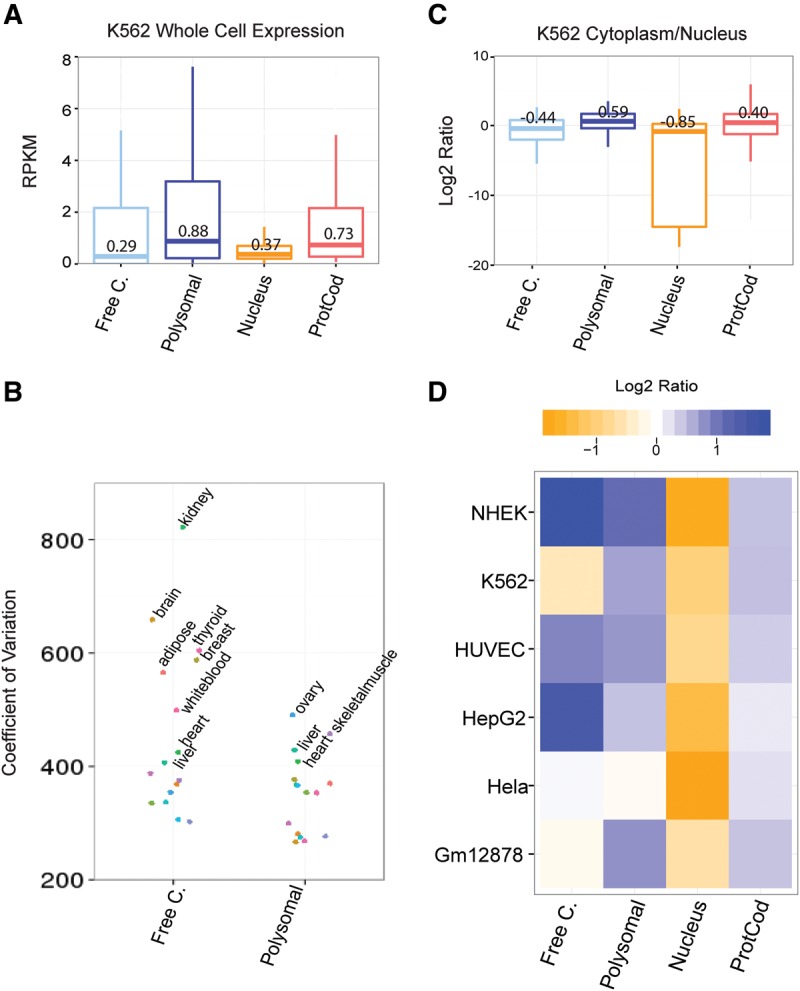
Expression of cytoplasmic and ribosome-associated lncRNAs in human tissues, cells, and subcellular fractions. (*A*) Expression in K562 whole cell by RNA-seq. Numbers indicate median value. (*B*) Coefficient of variation (CV) for free cytoplasmic and polysomal transcripts expression in each of the 16 Human Body Map tissues. (*C*) Log_2_ cytoplasmic/nuclear RPKM ratios calculated from ENCODE RNA-seq for indicated RNAs in K562 [whole cell, poly(A)^+^]. For protein-coding mRNAs (ProtCod), data are only shown for detected transcripts. Median values are shown. (*D*) Subcellular localization of lncRNA in different cell lines. Colors reflect median cytoplasmic/nuclear log_2_ RPKM values.

Using K562 RNA-seq data from ENCODE we compared steady-state expression levels between the cytoplasmic lncRNA classes. Polysomal lncRNAs have the highest median expression, significantly higher than free cytoplasmic and nuclear lncRNAs (*P* = 0.005/8 × 10^−6^/0.8 for free cytoplasmic/nuclear/protein-coding versus polysomal, Wilcoxon test), and exceeding even protein-coding mRNAs (Fig. 5A). A similar trend was observed in Human Body Map tissues (*P* = 0.005 Wilcoxon test, Supplemental Fig. S8A).

Polysomal and free cytoplasmic lncRNAs also display differences in expression variability. Some free cytoplasmic transcripts can achieve higher abundance in human tissues, but the percentage of transcripts expressed per tissue is lower (Supplemental Fig. S8B). As a result, variation across lncRNAs expression is higher for the free cytoplasmic group compared to the polysomal group, which tend to have a more homogeneous range of expression values in human tissues (Wilcoxon test: *P* = 0.02 for coefficient of variation, Fig. 5B; *P* = 3.3 × 10^−9^ for variance, data not shown).

Subcellular localization of lncRNA reported by polysome profiling is consistent with similar analysis using ENCODE RNA-seq (Djebali et al. 2012). Transcripts classified here as polysomal have significantly elevated cytoplasmic-nuclear ratios (Fig. 5C) (*P* = 3.7 × 10^−11^, Wilcoxon test), exceeding protein-coding mRNA. Free cytoplasmic RNA display a more balanced distribution between cytoplasm and nucleus. The subcellular localization of lncRNA observed in K562 is maintained across a variety of cell types (Fig. 5D). Once more, free and polysomal lncRNA gene sets have median cytoplasmic specificity exceeding that of protein-coding mRNAs (mean of medians across cell types: 0.50, 0.52, and 0.32 for free cytoplasmic, polysomal, and protein-coding sets, respectively).

### Evidence for conserved function of cytoplasmic lncRNAs

**FIGURE 6.**
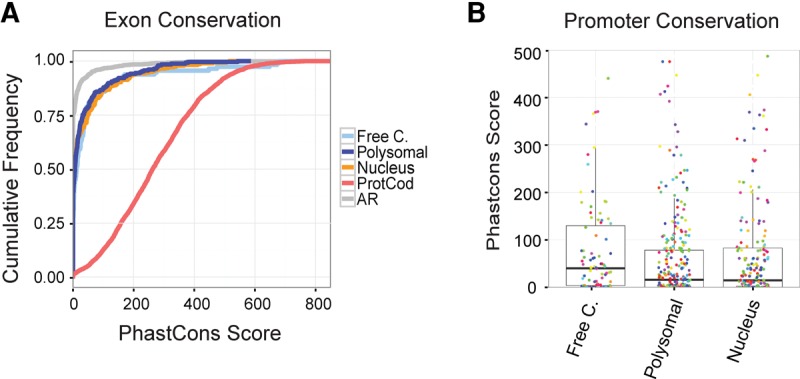
Ribosome-associated and cytoplasmic lncRNA are under purifying selection. (*A*) Cumulative distribution of the mean PhastCons nucleotide-level conservation for the exons of the indicated transcript classes. Scores for ancestral repeat (AR) regions are also included to represent neutral evolutionary rates. (*B*) Boxplot with overlaid dotplot comparing mean PhastCons nucleotide-level conservation of the promoters of each group of transcripts. When more than one transcript shares the same promoter, the value for the promoter is plotted only once. Each color represents a different gene and each dot a different transcript.

Purifying evolutionary selection represents powerful evidence for functionality. A number of studies have shown that lncRNAs are under weak but nonneutral purifying evolutionary selection (Ponjavic et al. 2007; Guttman et al. 2009; Derrien et al. 2012). We sought to test whether this holds true for cytoplasmic lncRNAs, and in particular whether different classes of cytoplasmic lncRNA described above might have experienced different strengths of selection. We extracted PhastCons measures of exonic and promoters conservation and compared lncRNAs of distinct subcellular origins (Fig. 6A). Ancestral repeats were treated as neutrally evolving DNA for comparison. As expected, protein-coding exons have highly elevated conservation. Free cytoplasmic, polysomal, and nuclear lncRNAs exhibit similar rates of nonneutral evolution.

Next, comparing PhastCons scores across promoters of expressed transcripts as a signal of expression regulation conservation, we found that promoters of free cytoplasmic transcripts are more conserved than those of polysomal (*P* = 0.06, Wilcoxon test) or nuclear transcripts (*P* = 0.01, Wilcoxon test) (Fig. 6B). This suggests that free cytoplasmic lncRNA promoters experience higher purifying evolutionary selection, consistent with a more conserved *cis* regulation and function.

### mRNA-like 5′ regions distinguish ribosomally bound lncRNAs

We next wished to identify factors that influence the recruitment of lncRNA to ribosomes. The most obvious feature is the presence of cryptic open reading frames (ORFs) that may serve as decoys for ribosomes. This is plausible given that lncRNAs contain abundant small ORF sequences (Dinger et al. 2008). In mRNAs, ORF length influences the number of ribosomes that can simultaneously bind, and hence the ribosomal fraction in which it sediments (compare ORF length for heavy and light polysomal mRNA in Supplemental Fig S9; van Heesch et al. 2014). However, for lncRNA we could find no evidence that ORFs determine ribosomal recruitment: Neither their total ORF coverage nor the length or coverage of their longest ORF correlates with ribosomal recruitment (Supplemental Figs. S10, S11). This is not surprising given that this lncRNA set was previously filtered by a variety of protein-coding prediction methods, which tend to use ORF length as a primary feature for prediction of protein-coding potential.

A number of other possible features distinguishing free and ribosome-associated lncRNAs were ruled out, including GC content (Supplemental Fig. S12), which clearly distinguishes coding and noncoding transcripts. We hypothesized that features known to influence mRNA recognition by ribosomes may also apply to lncRNA. For mRNAs, a number of factors control the scanning and engagement by ribosomes, including RNA structures within the 5′UTR and 7-methylguanylate capping (Jackson et al. 2010). We recently showed that splicing efficiency of lncRNAs is lower than mRNAs (Tilgner et al. 2012), but it does not distinguish ribosome-associated lncRNAs from other types (Supplemental Fig. S13).

**FIGURE 7.**
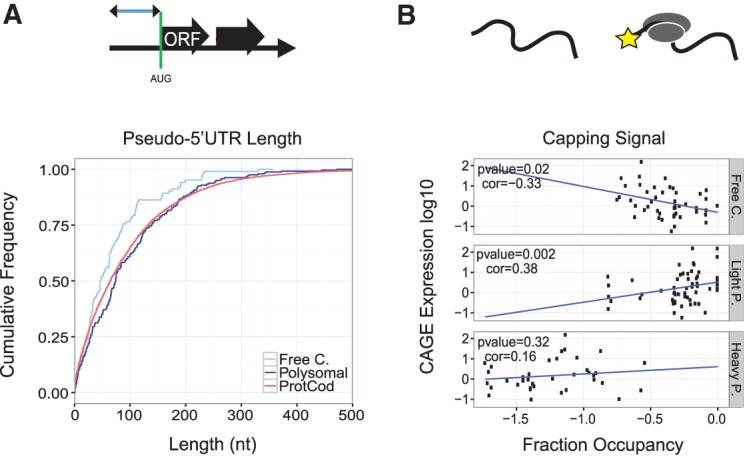
Ribosome-associated lncRNAs have mRNA-like 5′ ends. (*A*) The pseudo 5′UTR was defined as the nucleotide distance from the start to the first AUG trinucleotide (*top* row). Shown is the cumulative distribution of these lengths for each set of transcripts. (*B*) Capping efficiency was calculated by normalizing K562 cytoplasmic poly(A)^+^ CAGE tag expression to K562 cytoplasmic expression from RNA-seq data. For each fraction we plot the correlation between normalized CAGE expression and fraction occupancy for all transcripts detected in the fraction. Linear regression was used to assess the relationship between these variables. The cartoon depicts lncRNAs, with a yellow star denoting the 7-methylguanosine cap.

We next looked at the role of the 5′ end in ribosomal recruitment. Secondary structures in the 5′UTR have been shown to strongly influence translation of mRNAs (Kudla et al. 2009). While we do observe differences in the free energy folding of the first 50 nucleotides (nt) comparing mRNAs and lncRNAs, these differences disappear when we take into account variation in GC content (which may influence propensity of RNA folding) between mRNAs and lncRNAs. We do not see a clear overall disparity in structural propensity between ribosome-associated and free cytoplasmic transcripts either (Supplemental Fig. S14). Although lncRNAs do not have identifiable ORFs and hence 5′UTRs, they do contain abundant short “pseudo-ORFs”: random occurrences of in-frame start and stop codons. We defined the “pseudo-5′UTR” to be the region from the transcriptional start site to the first AUG trinucleotide of the first ORF (see Materials and Methods). Interestingly, the length of pseudo-5′UTRs does distinguish ribosome from non-ribosome-associated lncRNAs. Similar to protein-coding transcripts, polysomal lncRNA have significantly longer 5′UTR regions than free cytoplasmic (*P* = 0.003, Wilcoxon test), and similar to the 5′UTR for protein-coding genes (Fig. 7A). Thus long 5′UTR-like regions may contribute positively to ribosomal recognition of lncRNA.

Recognition of the 5′ methyl-guanosine cap is required for mRNA scanning by the 40S ribosomal subunit. Using CAGE (cap analysis of gene expression) data (Djebali et al. 2012), we investigated the relationship between the ribosomal recruitment of lncRNA and capping. Specifically, we examined the correlation between normalized CAGE signal and the relative concentration of lncRNA in cellular fractions. As shown in Figure 7B, there is a positive relationship between capping and recruitment to light polysomes, while a significant negative correlation is observed between capping and free cytoplasmic concentration. These data suggest that capping of lncRNA is a driver of ribosomal recruitment.

### Endogenous retroviral fragments are negatively correlated with ribosomal recruitment

**FIGURE 8.**
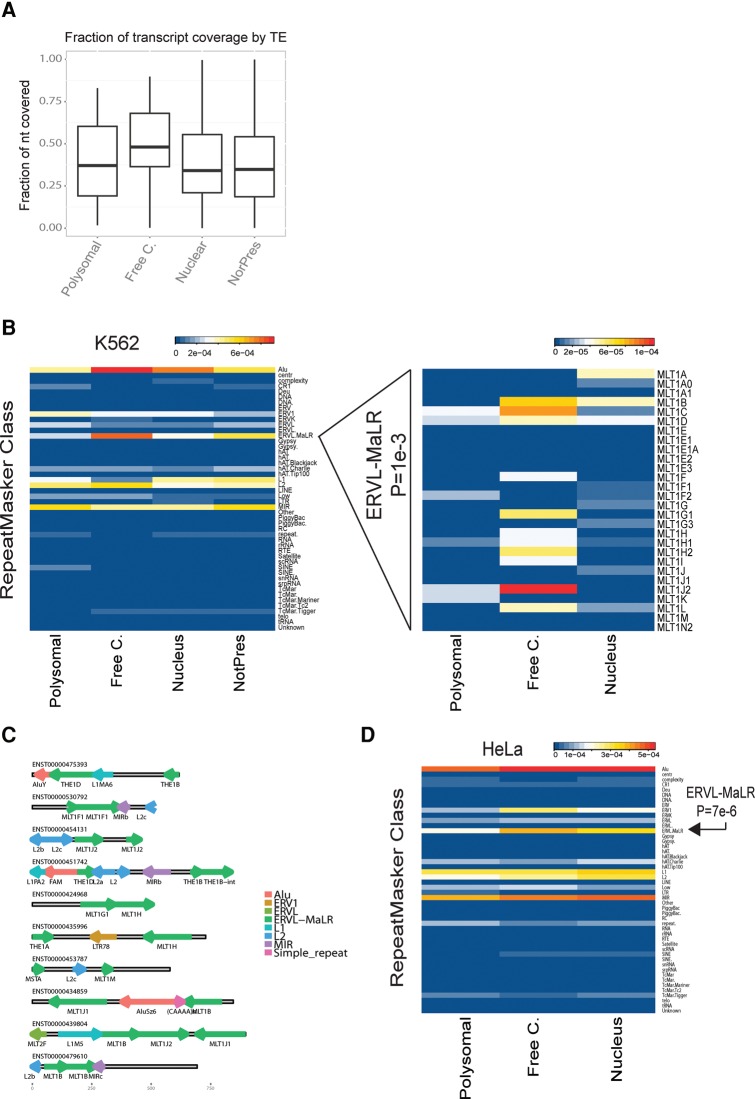
Transposable element composition of lncRNAs. (*A*) The fraction of each transcript covered by annotated transposable elements (TE) from RepeatMasker. (*B*) On the *left*, the heatmap shows the mean of the fractional overlap for RepeatMasker-defined classes, i.e., the nucleotide overlap by a TE of a lncRNA transcript, divided by the length of the transcript, averaged across all transcripts in a class. On the *right*, heatmap like previous one but showing data only for MLT-type repeats. (*C*) The repeat composition of a selection of free cytoplasmic, MLT-containing lncRNAs. The direction of the arrows indicates the annotated strand of the repeat with respect to the lncRNA. The colors represent the repeat class. (*D*) As in *B*, except showing data for HeLa derived from ribosome footprinting experiments (Ingolia et al. 2012).

There is growing evidence that transposable elements (TEs) contribute functional sequence to lncRNA (Kelley and Rinn 2012; Johnson and Guigó 2014). Taking all TE classes together, we observed an excess of TE-derived sequence within free cytoplasmic lncRNAs compared to polysomal (0.49 mean sequence coverage versus 0.39, *P* = 0.05, Wilcoxon test) (Fig. 8A).

We next investigated whether there exist TEs whose presence correlates with the subcellular localization of their host transcript. We calculated the insertion frequency of TE classes across lncRNAs, looking for cases with obvious differences between polysomal and free lncRNAs (see Materials and Methods). Figure 8B shows the nucleotide overlap, normalized for transcript length, for each TE across lncRNA classes. Similar results were found in equivalent analyses considering the frequency of TE insertions per nucleotide of transcript (data not shown). Supporting this approach, we observed the known relationship between the presence of Alu elements and elevated transcript expression: Alu elements are enriched amongst detected compared to undetected filtered lncRNAs (*P* = 4 × 10^−4^, hypergeometric test) (Kelley and Rinn 2012). Applying this analysis to all TE classes, we identified the endogenous retrovirus class ERVL-MaLR, which is approximately twofold enriched in free cytoplasmic lncRNAs compared to other expressed lncRNAs (*P* = 1.4 × 10^−3^, Wilcoxon test for insertion frequency) (Fig. 8B). Closer inspection revealed that this effect is not due to a single repeat type, but rather to around a dozen subclasses of MST, MLT, and THE endogenous retroelements (Fig. 8B). We found no significant difference in the length of ERVL-MaLR insertions between lncRNA classes (Supplemental Fig. S15). Rather it is the relative proportion of transcripts carrying an insertion that differs between groups. A selection of ERVL-MaLR containing lncRNAs are shown in Figure 8C.

Enrichment of ERVL-MaLR class elements in free cytoplasmic lncRNAs appears to be independent of cell type: Using ribosome footprinting data from HeLa (Ingolia et al. 2012), we observe that ERVL-MaLR class TEs are specifically depleted from ribosome-bound lncRNAs (Fig. 8D). Together these data suggest that endogenous retrovirus fragments can influence lncRNA trafficking in the cell.

### Stability levels of ribosome-associated lncRNAs are sensitive to ribosome-stalling drugs

**FIGURE 9.**
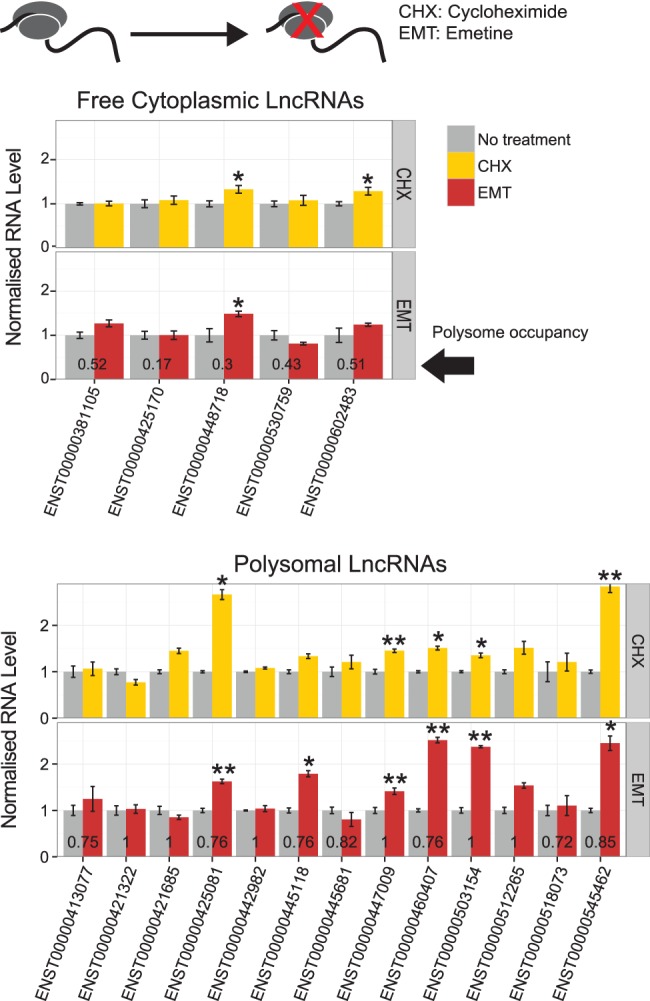
Changes in lncRNA stability in response to drug-induced ribosome stalling. K562 cells were treated with and without cycloheximide (CHX) or emetine (EMT), both treatments for blocking translation. Control and treated samples were then taken at 0 and 6 h after actinomycin D addition, which blocks transcription, and transcript levels were quantified in order to assess degradation rate of RNAs. Bars show mean fold change and standard deviation of three biological replicates (each performed in two technical replicates) of 6 h samples normalized to 0 h control samples. Treated samples were further normalized to control (untreated) samples. Bar numbers represent ratio from 0 to 1 of polysome occupancy for each transcript, according to microarray data (1 indicates transcript solely detected in light or heavy fractions, 0 for those undetected in either fraction). Results are shown separately for transcripts classified as free cytoplasmic or as polysomal transcripts. Statistical significance was calculated by one-sided *t*-test ([*] *P* < 0.05, [**] *P* < 0.01).

We next asked whether recruitment to ribosomes had any effect on lncRNA stability. It was proposed by Chew et al. (2013) that lncRNAs on the ribosomes are subject to degradation by the nonsense-mediated decay (NMD) pathway. Indeed, reports exist in human of ribosome-dependent degradation of snoRNA host genes (Makarova and Kramerov 2005; Lykke-Andersen et al. 2014), and this effect is reported to be widespread in plants (Kurihara et al. 2009) and for unannotated RNAs in yeast (Smith et al. 2014). Using the same candidate genes as before, we tested whether stalling of ribosomal elongation influenced lncRNA stability (Fig. 9). Using two distinct ribosome-stalling drugs, emetine (EMT) and cycloheximide (CHX), we observed consistent stabilization of lncRNAs after 6 h of treatment: Out of the six polysomal lncRNAs that respond to EMT, five also respond to CHX. Other transcripts were apparently unaffected under the conditions tested here. A panel of protein-coding mRNAs displayed a clearly different response: lower sensitivity in most cases (particularly to emetine), and even destabilization in response to ribosome-stalling in several cases (Supplemental Fig. S16). EMT/CHX-dependent stabilization was observed for two free cytoplasmic-classed lncRNAs, likely representing degradation of their nonnegligible subset of ribosome-bound transcripts. Together these data suggest that degradation of some cytoplasmic lncRNAs may be triggered by a translation-dependent mechanism.

## DISCUSSION

We have comprehensively and quantitatively mapped the ribosome-associated and cytoplasmic lncRNA populations of a human cell, discovering a substantial proportion of lncRNA associated with the translation machinery. This supports the idea that an important population of lncRNAs exists in the mammalian cytoplasm, including low but detectable amounts of lncRNA considered to be strictly nuclear, such as *XIST* and *MALAT1*. It may be speculated that cytoplasmic lncRNAs play nonnuclear roles including translational control, cellular metabolism, and signal transduction. Our findings, however, also suggest the possibility that degradation at the ribosome may be a general mechanism for the control of the cellular lncRNA population and the endpoint of the lncRNA lifecycle.

Polysome profiling appears to distinguish lncRNAs with distinct properties. We have attempted to rather crudely classify transcripts according to their fraction of maximum detection, but most transcripts are detected at varying concentrations in all fractions. Nevertheless, through this classification we have managed to discover features that distinguish lncRNAs and have laid a foundation for predicting lncRNA localization de novo. Similarly, a recent study discovered an RNA motif that predicts and appears to confer nuclear localization (Zhang et al. 2014). We find that lncRNAs localized in the light polysomal fraction tend to have mRNA-like 5′ features, more specifically a nonrandom long “pseudo-5′UTR” and the presence of a cap structure. This is consistent with the importance of 5′ recognition in the initiation of translation (Jackson et al. 2010), and a previous report showing that a cap structure is necessary for NMD of noncoding transcripts (Kurihara et al. 2009). Other mRNA-like features such as GC content or open reading frames do not appear to influence ribosomal interaction, at least in this data set.

In contrast, repetitive sequence features, and particularly human endogenous retrovirus fragments, are negatively associated with ribosomal recruitment. This is perhaps to be expected, given that mRNAs are depleted of such repeats compared to lncRNA (Kapusta et al. 2013). The mechanism by which hERV prevents lncRNA from ribosomal recruitment remains to be ascertained, although we proposed recently that such fragments may interact with protein complexes that could antagonize ribosomal binding (Johnson and Guigó 2014). In summary, these findings represent a starting point for discovering features that distinguish lncRNA classes and may eventually lead to useful models for predicting such classes.

Are lncRNAs directly engaged by ribosomes? Or do they simply reside within the ribosomal molecular complex? This question cannot be definitively answered by the polysome profiling approach used here, since in both cases we would expect lncRNA to co-sediment with polysomes. Nevertheless, two observations made here have bearing on this issue, and point to direct engagement: (i) stabilization of cytoplasmic lncRNAs in response to translation inhibitors cycloheximide and emetine; (ii) light polysomal lncRNAs are enriched for mRNA-like 5′ features known to be necessary for ribosomal engagement. These conclusions are also consistent with evidence from plants (Kurihara et al. 2009) and yeast (Smith et al. 2014). Furthermore, such engagement is consistent with the existence of ribosomal footprints in lncRNA observed previously (Ingolia et al. 2011).

Differences between light polysomal and heavy polysomal lncRNA may also shed light on this question. As we show for mRNAs, and was previously shown (van Heesch et al. 2014), mRNAs with longer ORFs tend to be more associated with heavy polysomes. We see a far lower association between lncRNA and heavy polysomes, compared to mRNA. If lncRNAs were indirectly bound to ribosomal complexes, then one might expect a stoichiometric relationship between polysome number and lncRNA concentration, i.e., a greater association with heavy compared to light polysomes. This is the opposite of what we observe. Furthermore, we do not observe correlation between capping and heavy polysomal recruitment, as we see for light polysomes. Together, all these arguments lead us to tentatively propose that, in the case of light polysomal lncRNA, we are observing at least a proportion of transcripts that are directly engaged by ribosomes. On the other hand, the far weaker heavy polysomal association observed for lncRNA may be due to indirect binding, since sufficiently large ORFs required for simultaneous binding of multiple ribosomes are absent.

If the above conclusion of direct engagement of lncRNAs by active ribosomes is correct, it raises the question of whether peptides are produced as a result. We do not find evidence for this, although our results do not rule it out either: The transcripts analyzed passed four distinct filters for protein-coding potential based on experimental mass spectrometry, evolutionary conservation, sequence composition, and similarity to known proteins. This makes it likely that if any peptides are translated from these sequences, they are degraded rapidly and have no selected function for the cell. It is also possible that lncRNAs are degraded rapidly after a single pioneer round of translation, limiting any translation to a single peptide molecule per RNA molecule. While it is likely that the predominant role of the majority of lncRNAs is not the production of a peptide, examples do exist of where noncoding RNAs do produce functionally important peptides (Ho et al. 2015).

Although it is tempting to propose that ribosome-associated lncRNAs regulate protein translation, we must also seriously consider an alternative possibility: that the ribosome represents a mechanism for cellular control of lncRNA levels. Considering that lncRNAs are thought to be principally regulatory molecules, this is consistent with the fact that regulatory proteins, and the mRNAs that encode them, tend to have short half-lives and high degradation rates (Yang et al. 2003; Schwanhäusser et al. 2011) required for temporally responsive gene networks. Indeed, it is perhaps not surprising that these mRNA-like transcripts—capped, polyadenylated and 100–10,000-nt long—should be recognized by the cell as mRNAs and trafficked accordingly. The emetine and cycloheximide data presented here lead us to hypothesize that, at least for a subset of cytoplasmic lncRNA transcripts, ribosomal recruitment results in degradation. The exact mechanism for this was not tested here, although an obvious candidate would be the translation-coupled nonsense-mediated decay (Lykke-Andersen et al. 2014). If NMD is responsible, then one testable hypothesis would be that single-exon transcripts (lacking the exon junction complexes required for NMD) should be unaffected. Future global analyses of lncRNA stability in response to loss of the NMD pathway should help clarify the importance of this degradation pathway.

In summary, these data support the notion that strictly nonribosomal cytoplasmic lncRNAs are the exception rather than the norm. Rather, cytoplasmic lncRNA molecules frequently find their way to the translational machinery. Stalling of the latter by drugs results in the stabilization of at least some of these lncRNAs. Thus, and in contrast to previous thinking, the RNA degradation-promoting activity of the ribosome may execute a crucial yet unconventional role as the final destination of cytoplasmic long noncoding RNAs.

## MATERIALS AND METHODS

### Polysome fractionation

For polysome fractionations, 20 million K562 cells were incubated with 100 µg/mL of cycloheximide (Sigma, Cat C4859) for 10 min. Cell pellets were resuspended in 200 µL RSB buffer (20 mM Tris–HCl, pH 7.4, 20 mM NaCl, 30 mM MgCl_2_, 200 µg/mL cycloheximide, 0.2 mg/mL heparin (Sigma, Cat No. H4787), 1000 unit/mL RNasin), then lysed with an equal volume of Lysis Buffer (1× RSB, 1% Triton X-100, 2% Tween-20, 200 µg/µl heparin) with (high stringency) or without (low stringency) 1% Na deoxycholate. Following incubation on ice for 10 min, extracts were centrifuged at 13,000*g* for 3 min to remove the nuclei. Supernatants were further centrifuged at 13,000*g* for 8 min at 4°C. Equal OD units were loaded onto 10%–50% linear sucrose gradients (prepared in 10 mM Tris–HCl pH 7.4, 75 mM KCl, and 1.5 mM MgCl_2_), and centrifuged at 36,000 rpm for 90 min at 8°C in a SW41 rotor (Beckman Coulter). Twelve fractions were collected from the top of the gradient using a piston gradient fractionator (BioComp Instruments). A UV-M II monitor (BIORAD) was used to measure the absorbance at 254 nm. Of note, 110 µL of 10% SDS and 12 µL of proteinase K (10 mg/mL Invitrogen) was added to each 1 mL fraction and incubated for 30 min at 42°C. Fractions 1–5, 6–8, and 9–11 were pooled corresponding to groups free cytoplasmic (free/monosomal), light polysomal (Light P.), and heavy polysomal (Heavy P.), respectively. For puromycin-treated samples, cells were incubated in 100 µg/mL puromycin for 15 min prior to processing and puromycin was used in place of cycloheximide in all the buffers.

Unfractionated cytoplasmic RNA and pooled polysomal RNAs were purified using phenol chloroform isoamyl extraction followed by LiCl precipitation to remove the heparin. The integrity of the samples was monitored by a Bioanalyzer. For qRT-PCR analysis equal volumes of RNA were used to synthesize cDNA using the Superscript III Reverse Transcriptase (Invitrogen) according to manufacturer's instructions. Two bacterial spike-in RNAs, Dap and Thr, were added before RNA purification to equal volumes of each polysomal RNA pool. Gene-specific primers were used with SYBR Green for qRT-PCR on an ABI PRISM 7900 Sequence Detection Systems. Candidate CT values were normalized to the spike in controls Dap and Thr that were present at equal concentrations per pool. Relative RNA levels are presented as a percentage of the RNA present in each pool with 100% RNA calculated as the sum of the FM, LP, and HP pools.

### Microarray design

This study was carried out using Agilent custom gene expression microarrays, in the 8 × 60k format with 60mer probes. Probes were designed using eArray software with standard settings: base composition methodology/60 mer/4 probes per target/sense probes/best probe methodology/3′ bias. Probes were designed for 14,700 transcripts from the entire Gencode v7 lncRNA catalogue, in addition to 26 known lncRNAs from www.lncrnadb.org (Amaral et al. 2011) and 90 randomly selected protein-coding housekeeping genes. The array was then filled with probes targeting 2796 randomly selected protein-coding gene probes. Microarray design details are available from the Gencode website (http://www.gencodegenes.org/lncrna_microarray.html).

### Microarray hybridization and probe filtering and quantification

For each sample, 100 ng of total RNA was labeled using Low Input Quick Amp Labeling kit (Agilent 5190-2305) following manufacturer's instructions, including the addition of standard spike-ins (Agilent One Color RNA Spike-In Kit, product number 5188-5282). mRNA was reverse transcribed in the presence of T7-oligo-(dT) primer to produce cDNA. cDNA was then in vitro transcribed with T7 RNA polymerase in the presence of Cy3-CTP to produce labeled cRNA. The labeled cRNA was hybridized to the Agilent SurePrint G3 gene expression 8 × 60K microarray according to the manufacturer's protocol. The arrays were washed, and scanned on an Agilent G2565CA microarray scanner at 100% PMT and 3-µm resolution. Intensity data were extracted using the Feature Extraction software (Agilent).

Using the Bioconductor package limma, raw data were taken from the Feature Extraction output files and corrected for background noise using the normexp method (Ritchie et al. 2007). To enable comparison across samples, we performed normalization between arrays using cyclic loess normalization based on spike-in RNAs at known concentrations, also using the limma package. After normalization we assessed the accuracy of the microarray data by plotting the log of the processed signal for each spike-in in each sample against the log of the known relative concentration (Supplemental Fig. S17). To ensure accurate estimation of lncRNA concentrations, we defined for each sample the intensity where the spike-in RNAs’ signal to concentration deviated from unity, and only considered probes above this level (Fig. 1A).

LncRNA transcripts and protein-coding genes were considered to be present in a sample when more than half of their probes were detected above the cutoff (protein-coding genes with only one probe were selected as present if its single probe was considered to be detected). The expression intensity value for transcripts or genes “present” was computed as the mean of its present probes. Variances in probe intensity values within probesets were significantly different when comparing all probesets from transcripts present in a sample (Levene's test). To avoid nonrepresentative intensity values, 5% of transcripts (for each sample) with highest probeset variance were excluded. Using linear regression from spike-in plots we computed log_10_ concentration values for all transcripts and genes present in each sample and multiplied its concentration by a factor in order to correct by the initial amount of RNA in each pooled group.

All statistical analyses were performed with the Bioconductor project (http://www.bioconductor.org/) in the *R* statistical environment (http://cran.r-project.org/) (Gentleman et al. 2004).

### Preparation of filtered lncRNA gene catalogs

We first filtered the former set to remove any transcripts that potentially result from misannotated extensions or isoforms of protein-coding genes or pseudogenes. Any gene was discarded that has at least one transcript fulfilling one of the following conditions: overlapping on the same strand a Gencode v18 annotated pseudogene, overlapping on the same strand an exon of a protein-coding mRNA, or lying within 5 kb and on the same strand as an Gencode v18 protein-coding transcript or pseudogene (1140 transcripts, 517 genes). This resulted in a data set of 13,358 lncRNA transcripts (8615 genes). Next, genes having at least one transcript predicted as protein-coding by at least one method, were classified as “potential protein-coding RNAs” (4350 transcripts, 1867 genes), while the remainder were classified as “filtered lncRNAs.” The four filtering methods used were: (i) PhyloCSF, a comparative genomics method based on phylogenetic conservation across species (Lin et al. 2011). The analysis was performed using 29 mammalian nucleotide sequence alignments and assessing the three sense frames. The alignment of each transcript was extracted from stitch gene blocks given a set of exons from Galaxy (Goecks et al. 2010). Transcripts with score >95 were classified as potential protein-coding, following the work of Sun et al. (2013). (ii) Coding Potential Assessment Tool (CPAT) (Wang et al. 2013), using the score threshold of 0.364 described by the authors. (iii) Coding Potential Calculator (CPC), a support vector machine-based classifier based on six biological sequence features, using a cutoff of 1 (Kong et al. 2007). (iv) Peptides: We used experimental mass spectrometry tag mappings from Pinstripe to identify any transcripts producing peptides (Gascoigne et al. 2012). Any transcript having an exonic, same strand tag mapping were designated as “potential protein-coding.” Collectively, sequence filters reduced the pool of analyzed transcripts to 9008 transcripts (6748 genes). The full table of classification data for all Gencode v7 lncRNA is available in Supplemental Table S1.

Applying these filters we define 345 filtered lncRNAs (205 genes), 374 potential protein-coding transcripts (145 genes), and 1130 protein-coding genes that are detected in K562 cytoplasm.

### Classification of array transcripts

From the polysome profiling analysis, detected lncRNAs and mRNAs were classified according to the microarray sample (condition) where they displayed the highest transcript-level signal. Thus, present transcripts were classified into heavy polysomal (Heavy P.), light polysomal (Light P.), and free cytoplasmic transcripts (free C.) transcripts. Given the low number of heavy polysomal lncRNAs, we pooled light polysomal and heavy polysomal lncRNAs into a single “polysomal” transcript class. The remaining protein-coding genes, which were not present in any microarray condition, were considered not present. Remaining filtered lncRNA transcripts were subsequently checked in ENCODE K562 nucleus RNA-seq. Those detected (defined as RPKM bio-replicates mean > 0 and IDR < 0.1) were classified as nuclear specific transcripts (nucleus). Remaining transcripts, which are not present in cytoplasm or in the nucleus are considered not present (NotPres).

### Peptide expression analysis

To estimate levels of peptides arising from mRNAs detected by this study, we downloaded supplemental data from Geiger et al. (2012). We estimated peptides’ expression by computing the mean of peptide intensity after iBAQ normalization and after label-free quantification (LFQ) of three K562 biological replicates. We defined the translation index as the ratio of peptide expression to mRNA concentration (the latter defined as the sum of concentrations in the three cytoplasmic fractions). We only considered peptides with expression above 0.5 (in log_10_ scale).

### RNA fluorescent in situ hybridization (FISH)

LncRNA probes were designed and synthesized by Biosearch Technologies. As a cytoplasmic positive control we used the protein-coding gene *GAPDH*, for which Stellaris FISH Probes (Biosearch Technologies) were commercially available. RNA FISH experiments were performed on HeLa cells following Stellaris RNA FISH protocol for adherent cells. Imaging was performed using an inverted fluorescent microscope.

### Cytoplasmic-nuclear localization using RNA-seq data

Mapped and quantified cytoplasmic and nuclear poly(A)^+^ RNA-seq data from six different cell lines (K562, HeLa, NHEK, HepG2, GM12878, HUVEC) were obtained from ENCODE (Djebali et al. 2012). Data were mapped using STAR software (Dobin et al. 2013) and processed with the FluxCapacitor (Montgomery et al. 2010) for transcript quantification. For each cell line we calculated cytoplasmic-nuclear RPKM ratios for transcripts detected in both that cell line and K562. RPKM was calculated as the mean of two available technical replicates, and only transcripts with mean > 0 RPKMs and IDR < 0.1 were considered present. We calculated log_2_ ratios of cytoplasmic expression versus nuclear expression (RPKM units) for those transcripts present in both nucleus and cytoplasm.

### Tissue expression analysis

We extracted tissue expression values for 16 human tissues from Human Body Map (HBM) RNA-seq data, downloaded from ArrayExpress under accession number E-MTAB-513. These data were used to quantify GENCODE v7 transcripts using the GRAPE pipeline (Knowles et al. 2013). This pipeline uses GEM mapper (Marco-Sola et al. 2012) and FluxCapacitor for transcript quantification.

### Conservation analysis

We extracted PhastCons scores from vertebrate species alignments and defined exonic and promoter average conservation. When comparing promoter conservation between groups, only one transcript per promotor was selected. If more than one transcript had the same promoter the value for the promoter was counted only once.

### Transposable element analysis

The 2013 version of RepeatMasker human genomic repetitive element annotations were downloaded from UCSC Genome Browser and converted to BED format. Using the tool IntersectBED, we calculated (i) the number of instances of intersection, and (ii) the number of nucleotides of overlap, between each lncRNA transcript and each transposable element. For each lncRNA transcript, this value was then divided by the nucleotide length of the transcript. Finally, the mean of these values was calculated across all transcripts in each class. Only the single transcript with highest exon count was considered from each gene, to avoid bias from lncRNA genes with numerous transcripts. We observed similar results using intersection frequency and nucleotide overlap analyses. This analysis was carried out for both transposable element types, and transposable element classes.

In order to test if the same trends are present when using cytoplasmic classifications from another cell line, we downloaded public data for HeLa cells and divided our filtered transcripts into three groups: polysomal, free cytoplasmic, and nuclear. We downloaded raw RNA-seq and footprinting reads from NCBI Gene Expression Omnibus (GEO) under accession numbers GSM546920 and GSM546921, and through GRAPE pipeline we aligned them to Human Genome version 19 (hg19) using GEM mapper. We used FluxCapacitor and GENCODE v7 exons annotation for quantification. We selected filtered and cytoplasmic transcripts from K562 present in HeLa cytoplasmic RNA-seq from ENCODE (Djebali et al. 2012), and defined as HeLa ribosome-associated transcripts those having >0 footprinting mappings. Based on ENCODE HeLa cytoplasmic and nuclear RNA-seq data, remaining lncRNAs having less than mean RPKM < 0.01 in either fraction were discarded. Remaining lncRNAs were divided according to the cytoplasmic/nuclear ratio as free cytoplasmic (with cytoplasmic RPKM > nuclear RPKM) and nuclear (when nuclear RPKM > cytoplasmic RPKM). Then, the same TE analysis was applied for these classifications.

### ORF analysis

We mapped all possible canonical open reading frames (ORFs) in each of six frames in lncRNA and protein-coding transcripts from Gencode. If more than one start codon was in frame with a stop codon, only the start codon for the longest ORF was considered. “Pseudo-5′UTRs” were defined as the nucleotide sequences from the transcription start site to the first AUG trinucleotide, for both protein-coding transcripts and lncRNAs.

### CAGE analysis of lncRNA capping

5′ cap analysis was performed on cap analysis gene expression (CAGE) tags from ENCODE (Djebali et al. 2012) for K562 cytoplasmatic poly+ RNA, and we mapped these tags to the microarray region comprising between 100 nt before and after transcription start sites of lncRNA. In order to assess the relationship between cytoplasmic class and capping, we compared CAGE tag presence (normalized by ENCODE K562 cytoplasmic poly+ RNA expression) to fractional occupancy in each class. The latter was calculated for each fraction by subtracting fraction input cytoplasmic log_10_ microarray concentration values from the sum of the three polysome profiling fractions concentration values (free cytoplasmic, light and heavy polysomal). Linear regression was performed to assess the relationship between CAGE tag presence and occupancy.

### RNA stability assay

Experiments were performed in biological triplicates. K562 cells were pretreated for 2 h with the drug (Cycloheximide CHX or Emetine EMT, both at 100 µg/mL), prior to addition of Actinomycin D (ActD, at 5 µg/mL) to block transcription. Control cells were treated identically, except that neither CHX nor EMT was added. Samples were taken at 0 and 6 h of ActD treatment, and the latter were normalized to the former. As there is no transcription in the presence of Act D, the decrease in RNA level between 0 and 6 h is indicative of the degradation rate of that mRNA. RNA was purified using TRIzol and Qiagen RNeasy columns. One microgram of RNA was used to make cDNA using RevertAid H Minus reverse transcriptase. Luminaris Color HiGreen High ROX qPCR master mix was used with gene specific primers for qRT-PCR on an ABI PRISM 7900 Sequence Detection Systems. Expression levels were normalized to the housekeeping gene GAPDH by the Δ–Δ Ct method. The mean of two technical replicate PCR results was calculated for each biological replicate.

## SUPPLEMENTAL MATERIAL

Supplemental material is available for this article.

## Supplementary Material

Supplemental Material
