# Supplementary material for: Cytoplasmic long noncoding RNAs are frequently bound to and degraded at ribosomes in human cells
Source: RNA. 2016 Jun;22(6):867–82. doi: 10.1261/rna.053561.115 (PMC4878613; doi:10.1261/rna.053561.115)
Supplement: Supplemental Material [file supp_22_6_867__index.html]

Cytoplasmic long noncoding RNAs are frequently bound to and degraded at ribosomes in human cells — Supplemental Material 

# Cytoplasmic long noncoding RNAs are frequently bound to and degraded at ribosomes in human cells

## Supplemental Material

**Files in this Data Supplement:**

- Supp\_Figures.pdf - pdf file
- Supp Legends.doc
- Supp\_TableS1.txt - txt file
