# Supplementary material for: Cytoplasmic long noncoding RNAs are frequently bound to and degraded at ribosomes in human cells
Source: RNA. 2016 Jun;22(6):867–82. doi: 10.1261/rna.053561.115 (PMC4878613; doi:10.1261/rna.053561.115)
Supplement: Supplemental Material [file supp_053561.115_Supp_Figures.pdf]

Figure S1

mRNAs

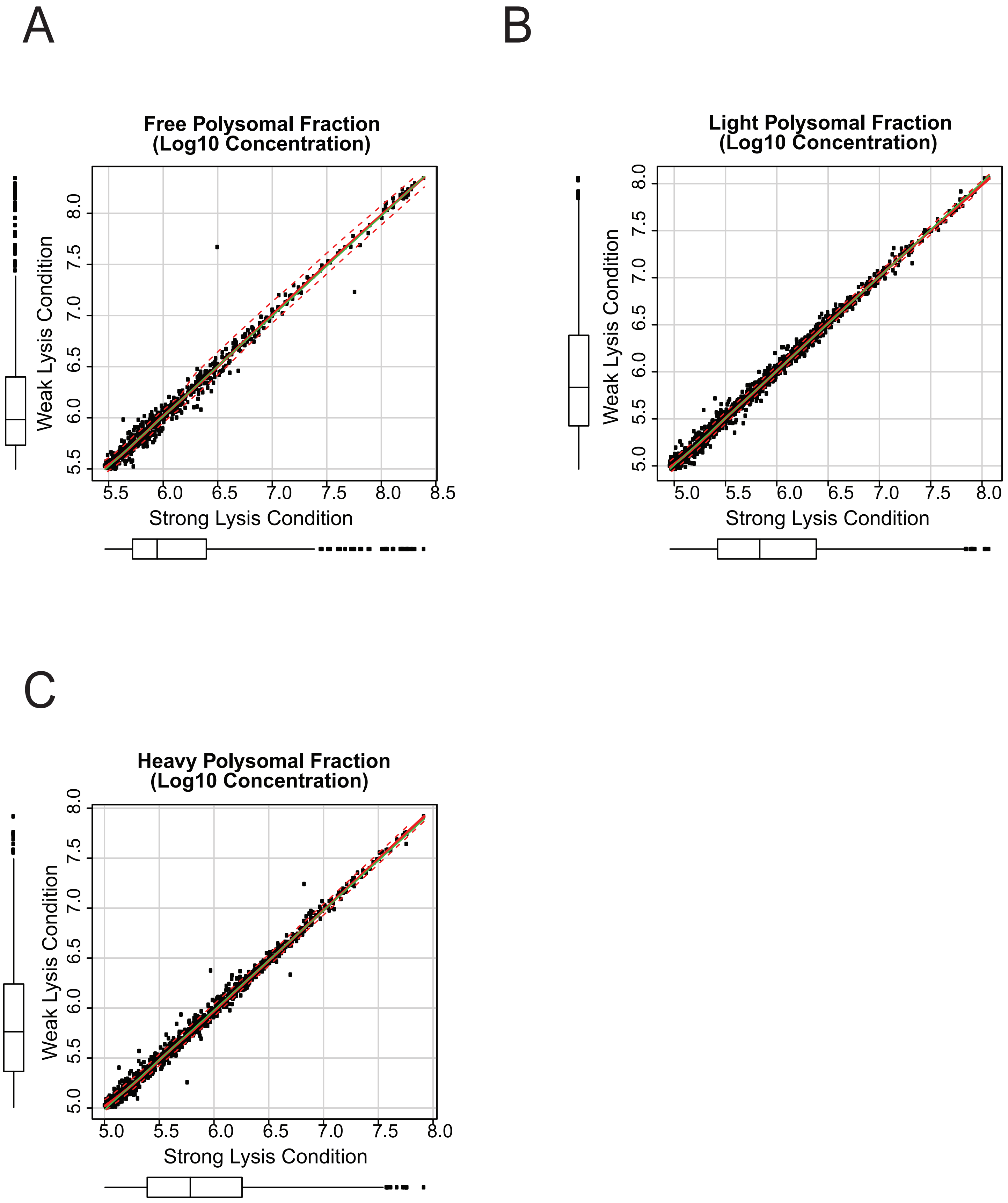

Figure S2

LncRNAs

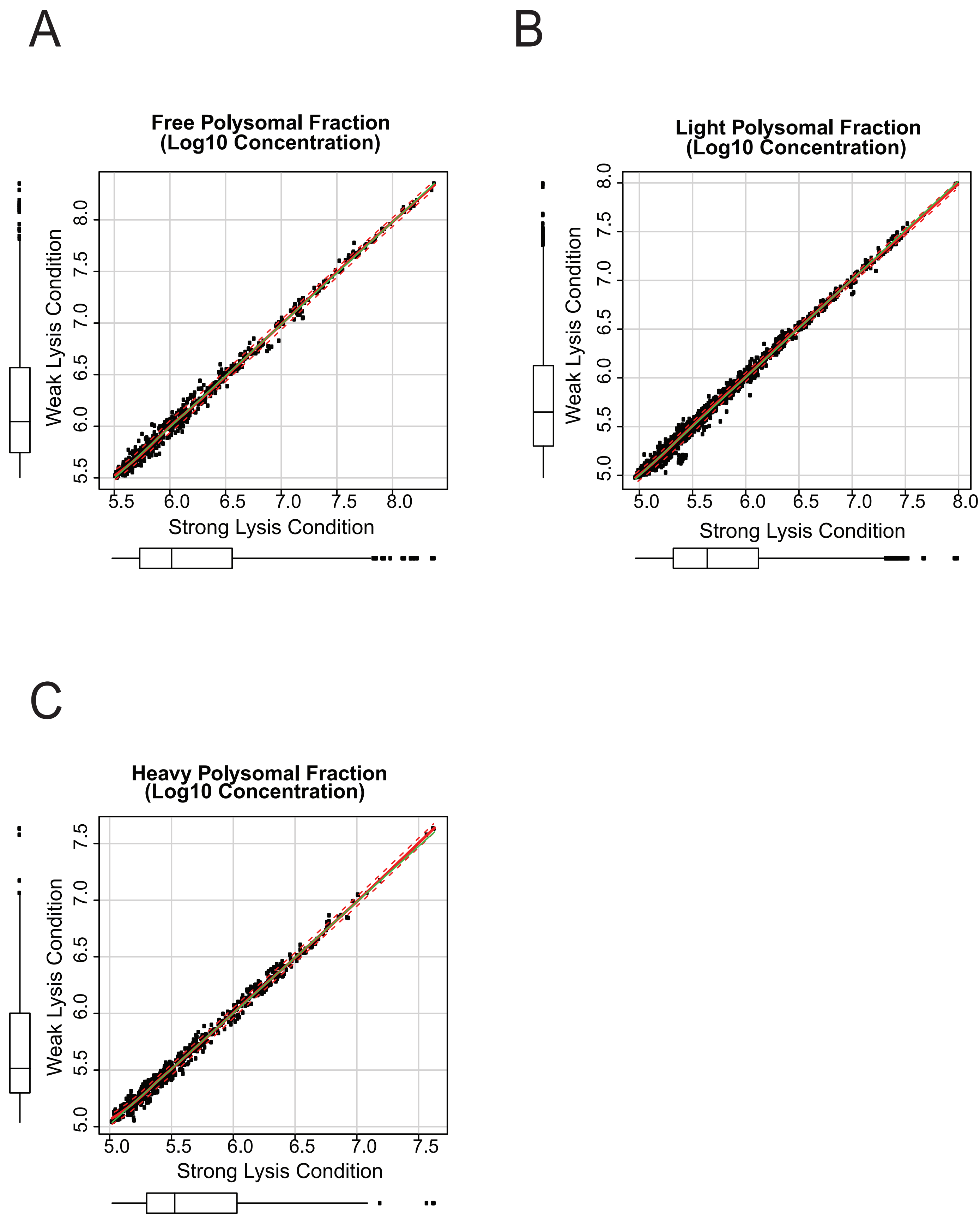

Figure S3

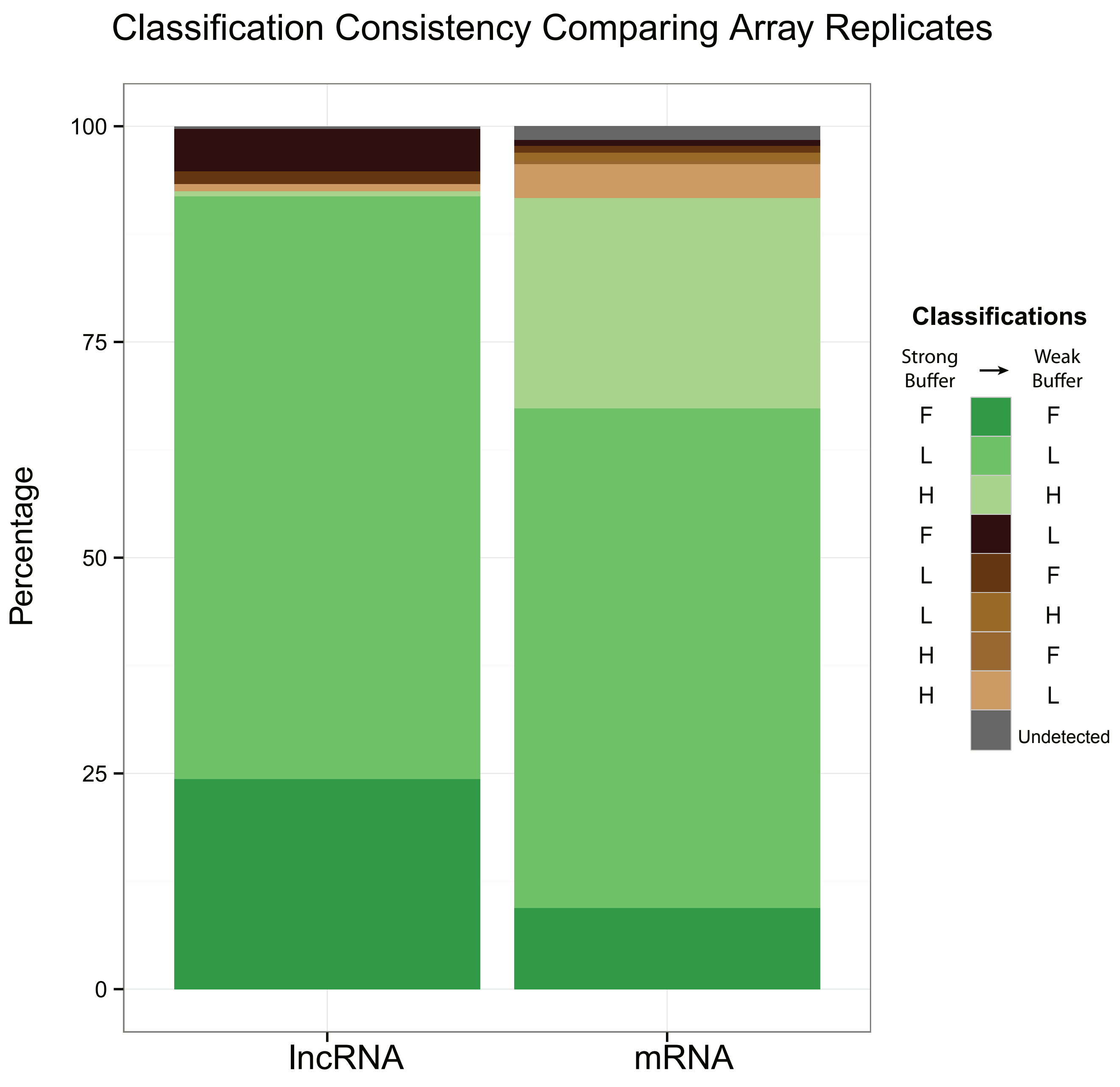
Classification Consistency Comparing Array Replicates
Percentage
100
75
50
25
0
lncRNA
mRNA
Classifications
Strong Buffer
Weak Buffer
F F
L L
H H
F L
L F
L H
H F
H L
Undetected

Figure S4

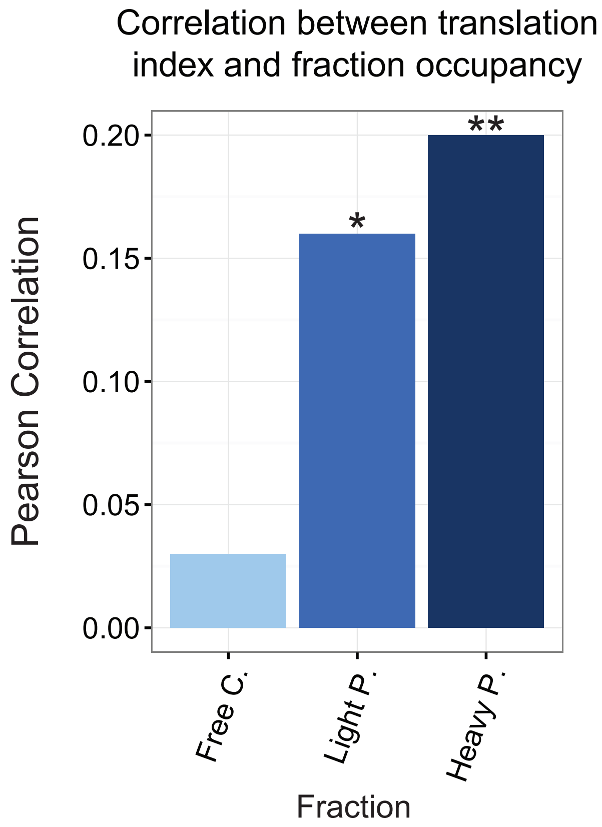

Correlation between translation index and fraction occupancy
Pearson Correlation
0.20
0.15
0.10
0.05
0.00
*
**
Free C.
Light P.
Heavy P.
Fraction

Figure S5

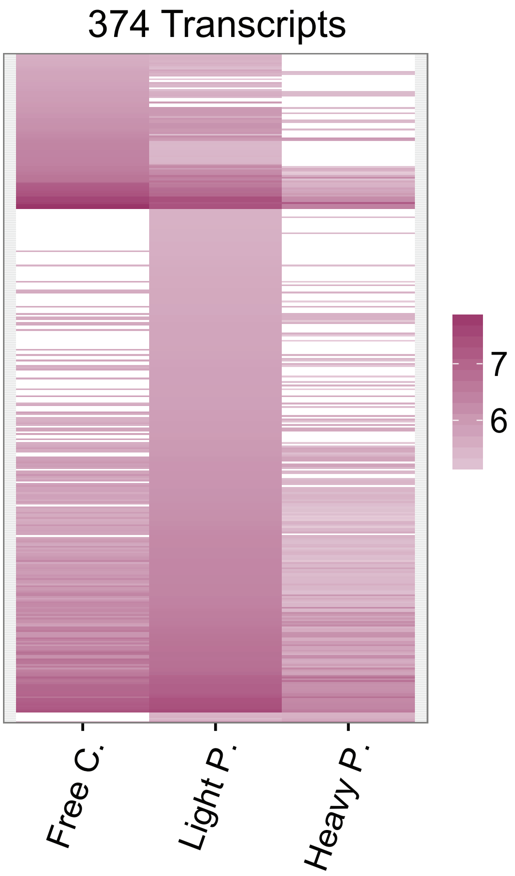

Figure S6

lncRNA MALAT1

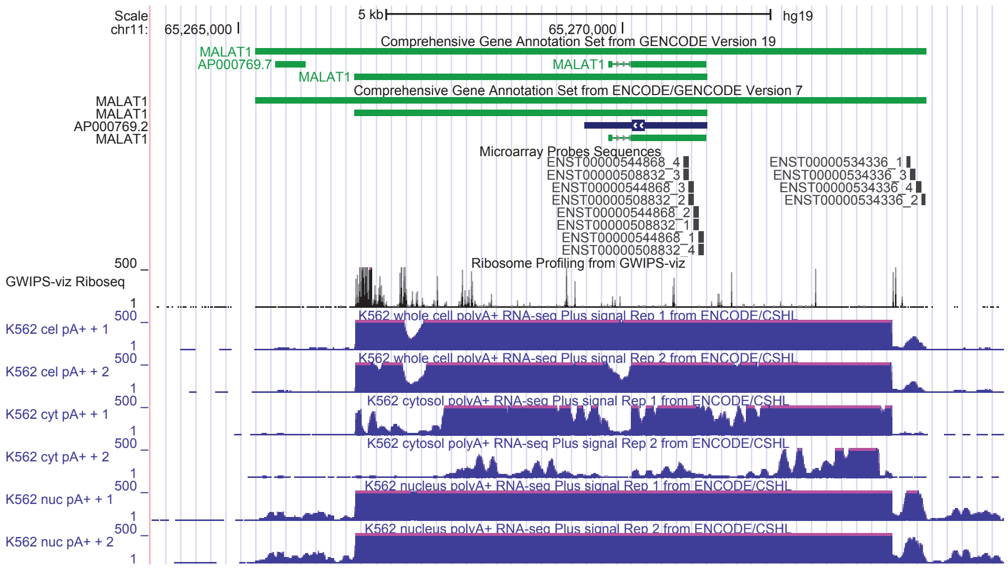

lncRNA XIST

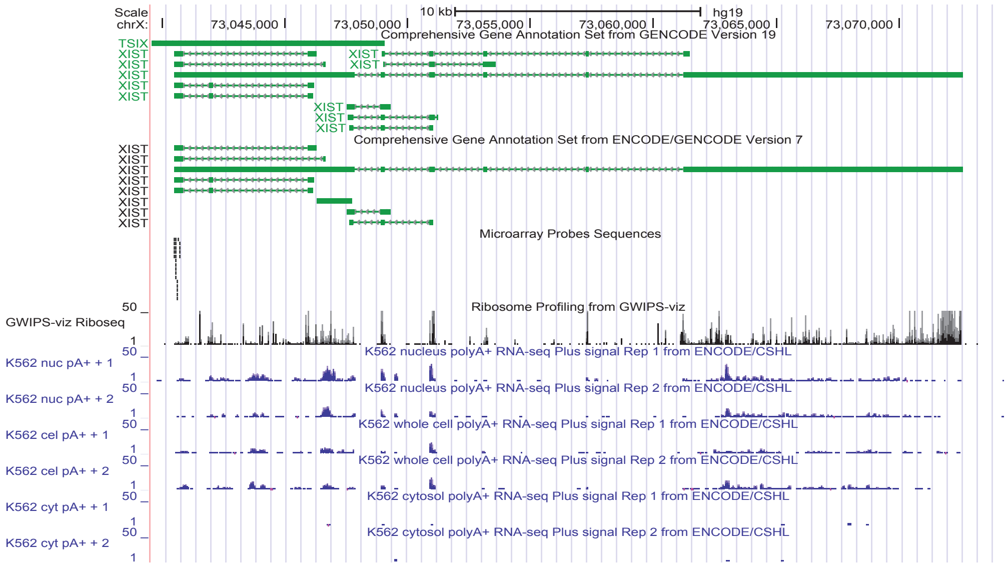

mRNA GAPDH

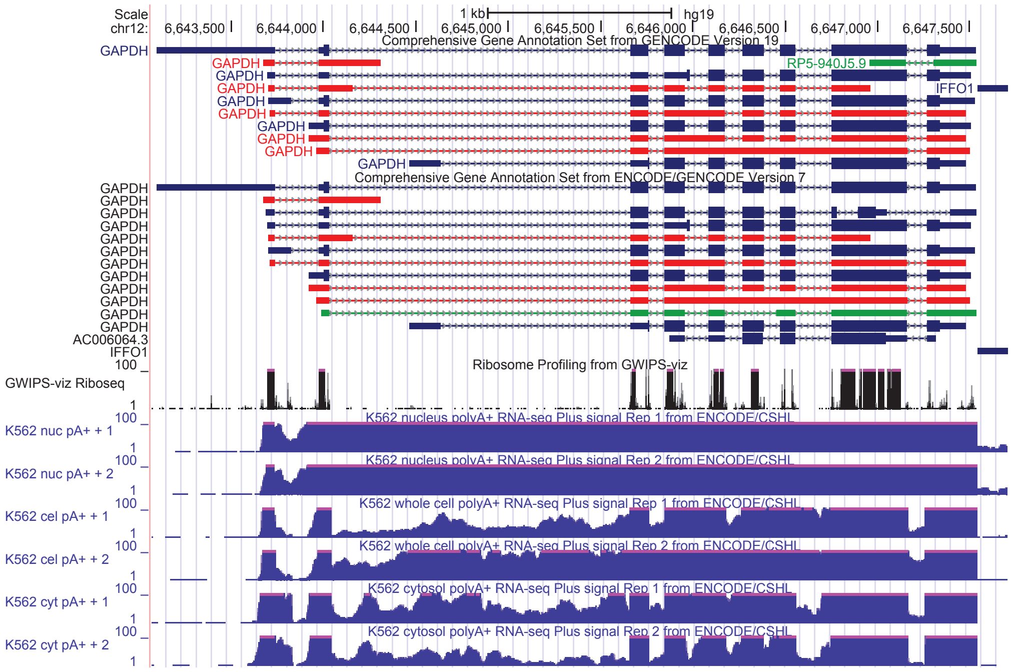

Figure S7

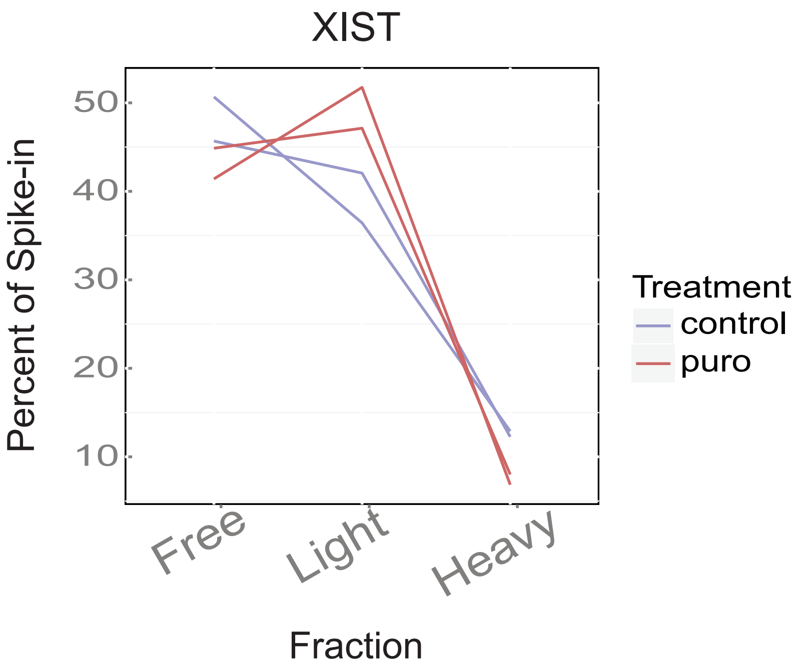

XIST
Percent of Spike-in
50
40
30
20
10
Free
Light
Heavy
Fraction
Treatment
control
puro

Figure S8

A

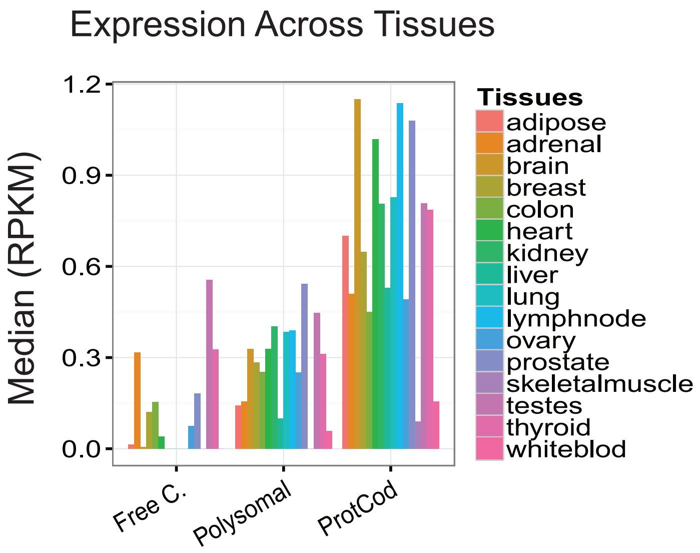

Expression Across Tissues
Median (RPKM)
1.2
0.9
0.6
0.3
0.0
Free C.
Polysomal
ProtCod
Tissues
adipose
adrenal
brain
breast
colon
heart
kidney
liver
lung
lymphnode
ovary
prostate
skeletalmuscle
testes
thyroid
whiteblod

B

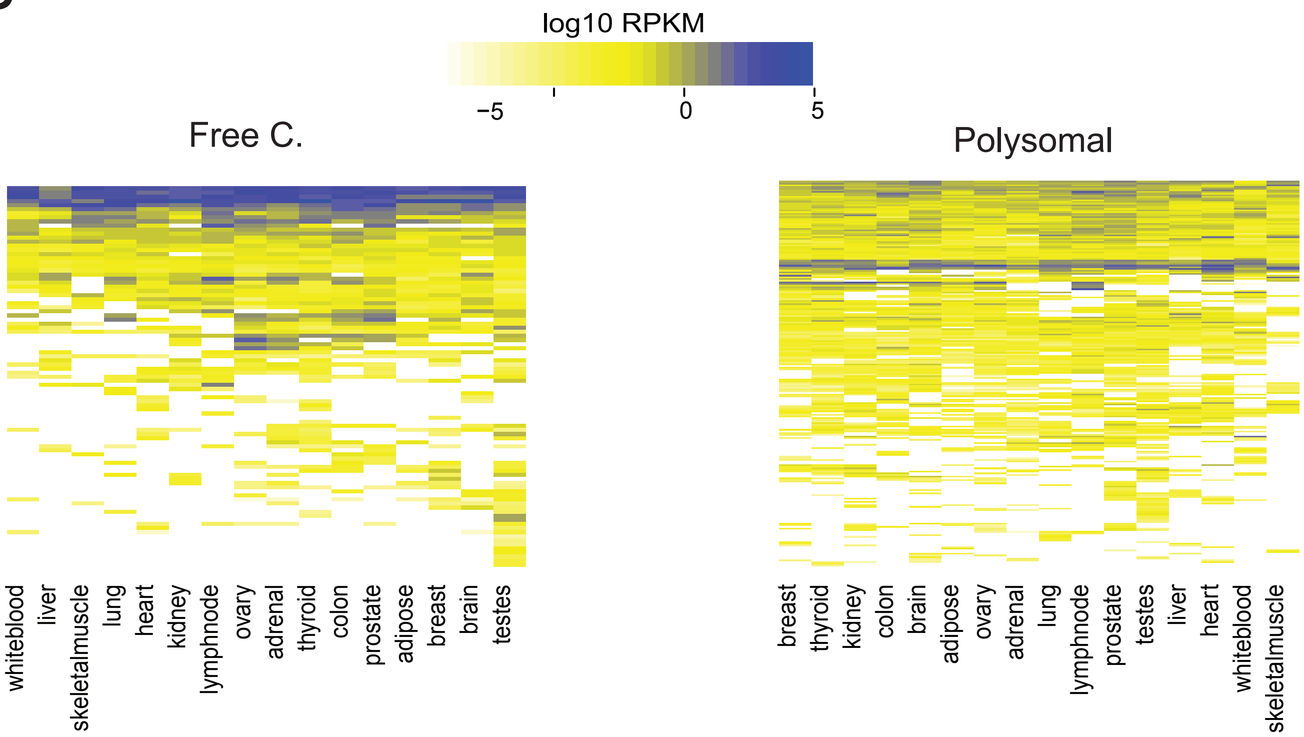

log10 RPKM
−5
0
5
Free C.
Polysomal
whiteblood
liver
skeletalmuscle
lung
heart
kidney
lymphnode
ovary
adrenal
thyroid
colon
prostate
adipose
breast
brain
testes
breast
thyroid
kidney
colon
brain
adipose
ovary
adrenal
lung
lymphnode
prostate
testes
liver
heart
whiteblood
skeletalmuscle

Figure S9

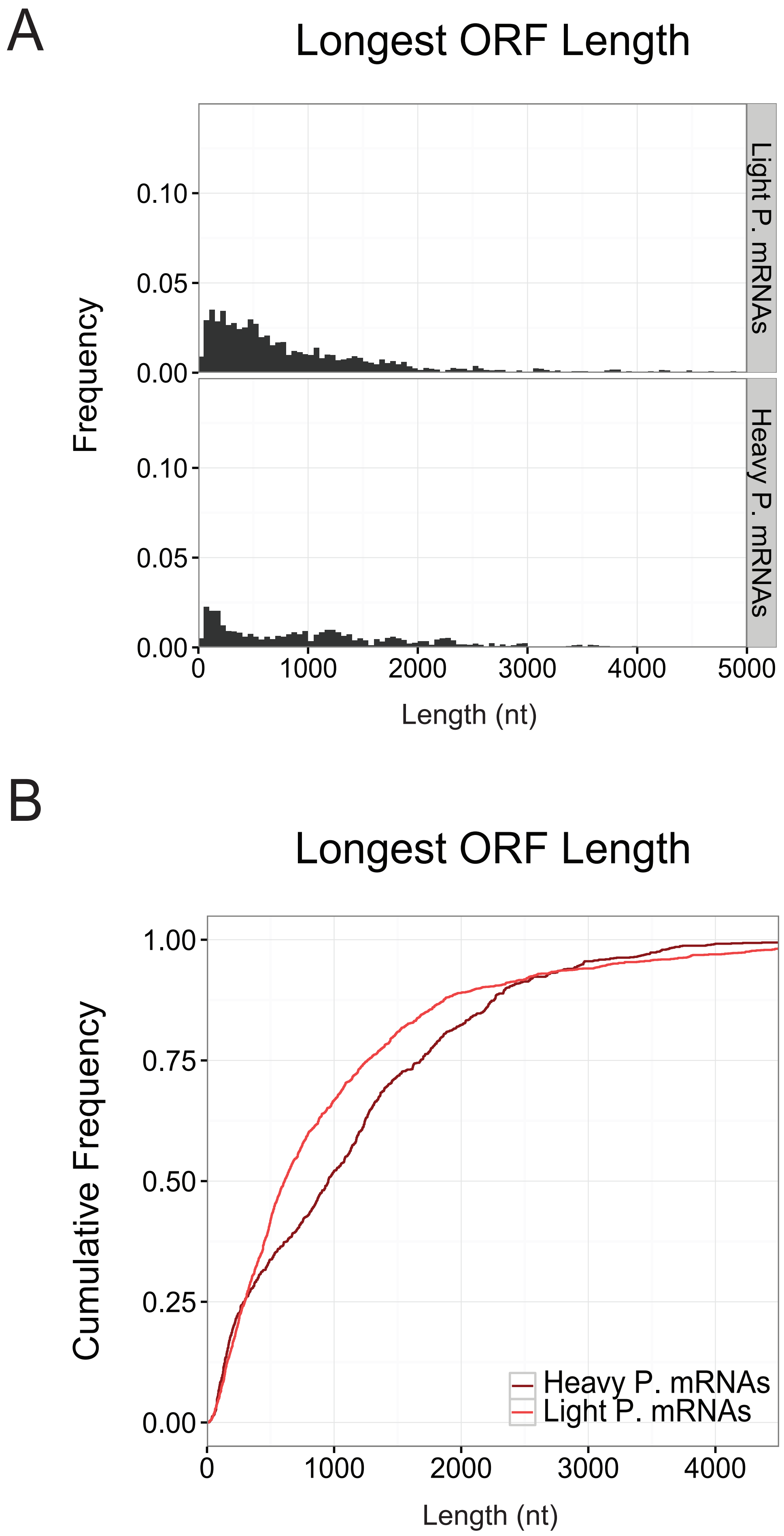
A
Longest ORF Length
Frequency
0.10
0.05
0.00
Light P. mRNAs
Heavy P. mRNAs
0
1000
2000
3000
4000
5000
Length (nt)
B
Longest ORF Length
Cumulative Frequency
1.00
0.75
0.50
0.25
0.00
Heavy P. mRNAs
Light P. mRNAs
0
1000
2000
3000
4000
Length (nt)

Figure S10

A

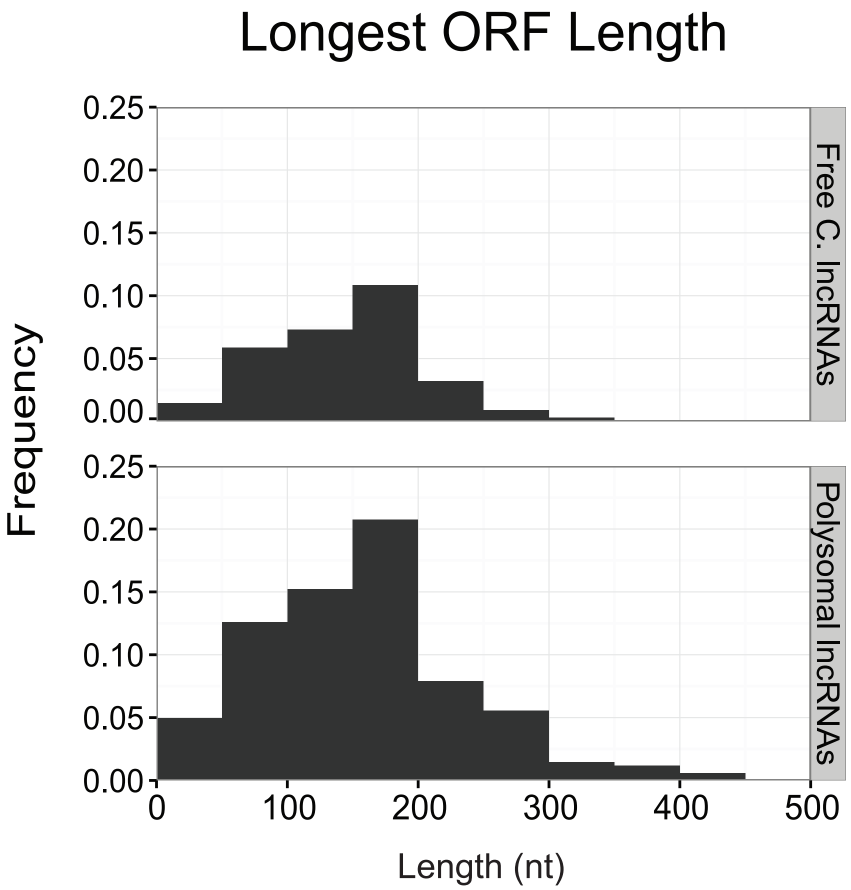

B

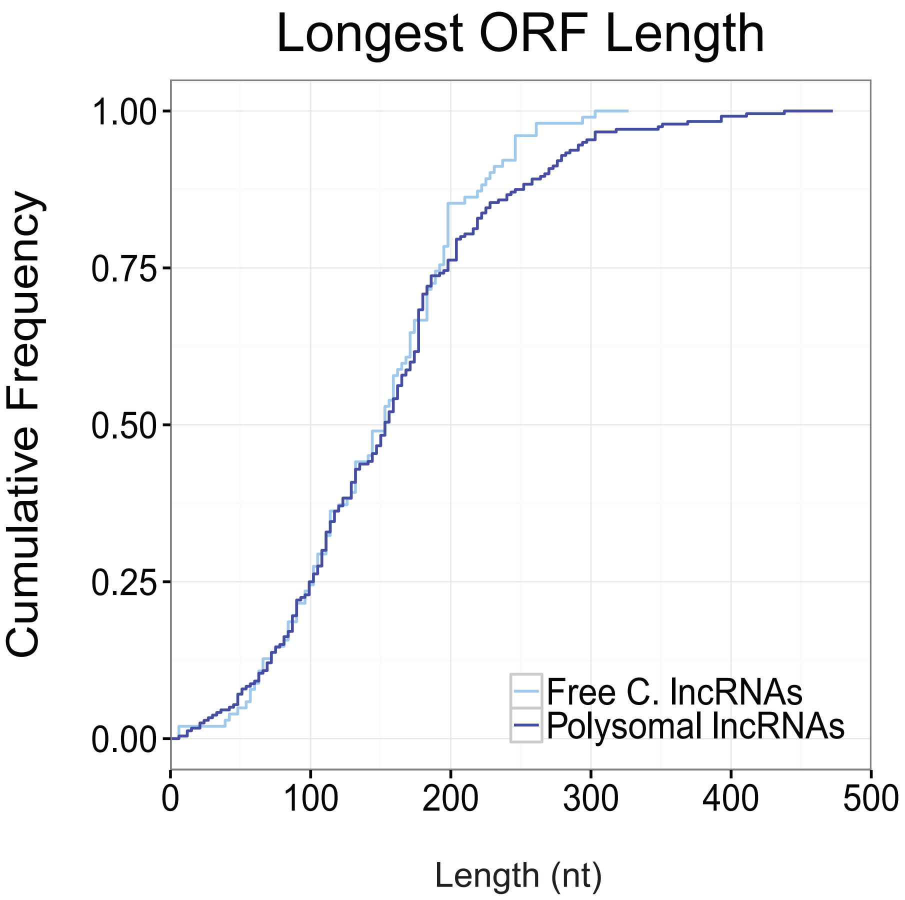

Figure S11

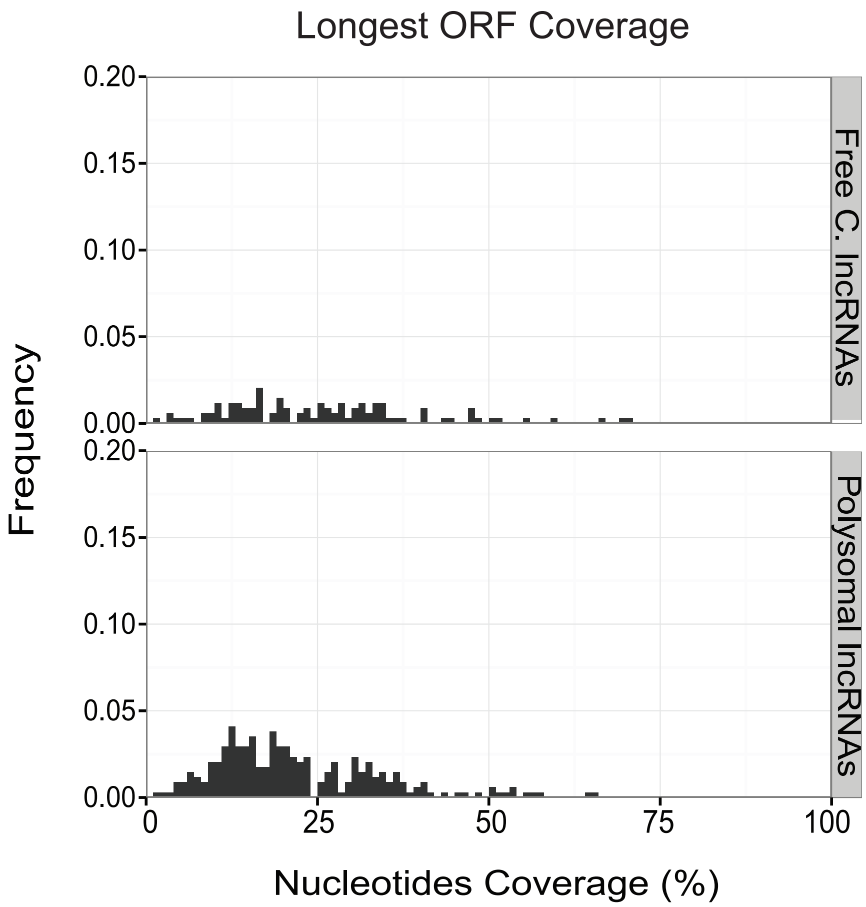

Longest ORF Coverage
Frequency
0.20
0.15
0.10
0.05
0.00
Free C. lncRNAs
Polysomal lncRNAs
0
25
50
75
100
Nucleotides Coverage (%)

Figure S12

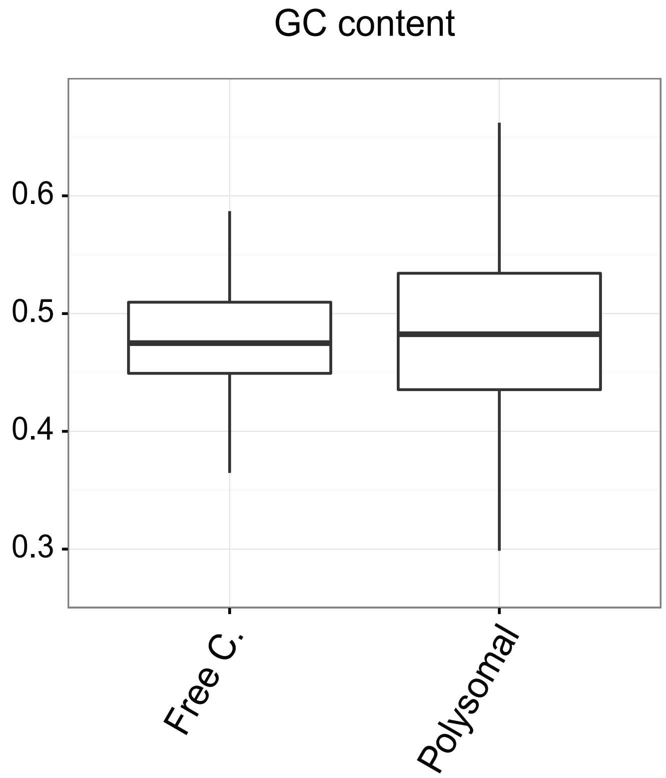

Figure S13

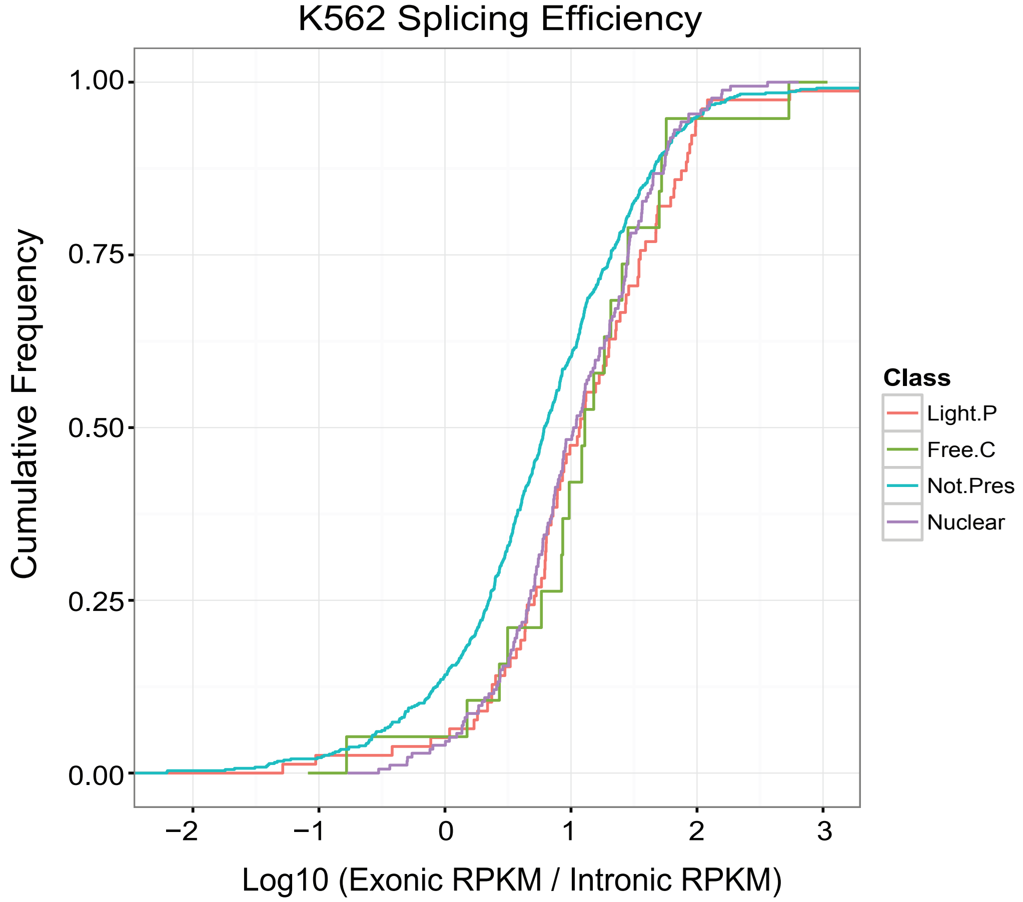
K562 Splicing Efficiency
Cumulative Frequency
1.00
0.75
0.50
0.25
0.00
−2
−1
0
1
2
3
Log10 (Exonic RPKM / Intronic RPKM)
Class
Light.P
Free.C
Not.Pres
Nuclear

Figure S14

A

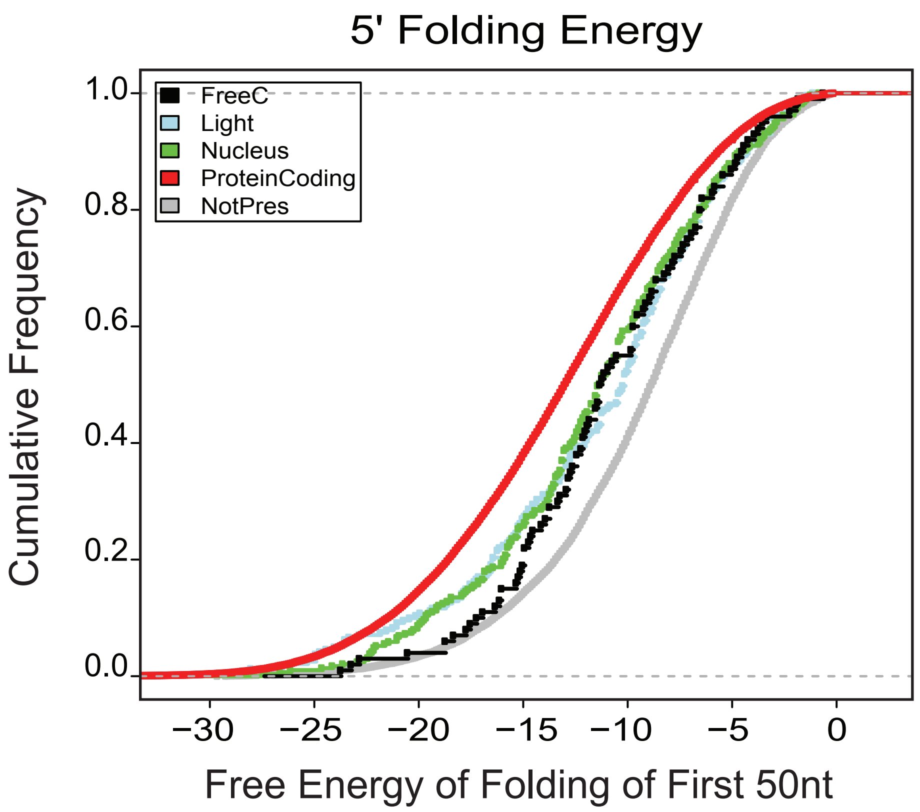

B

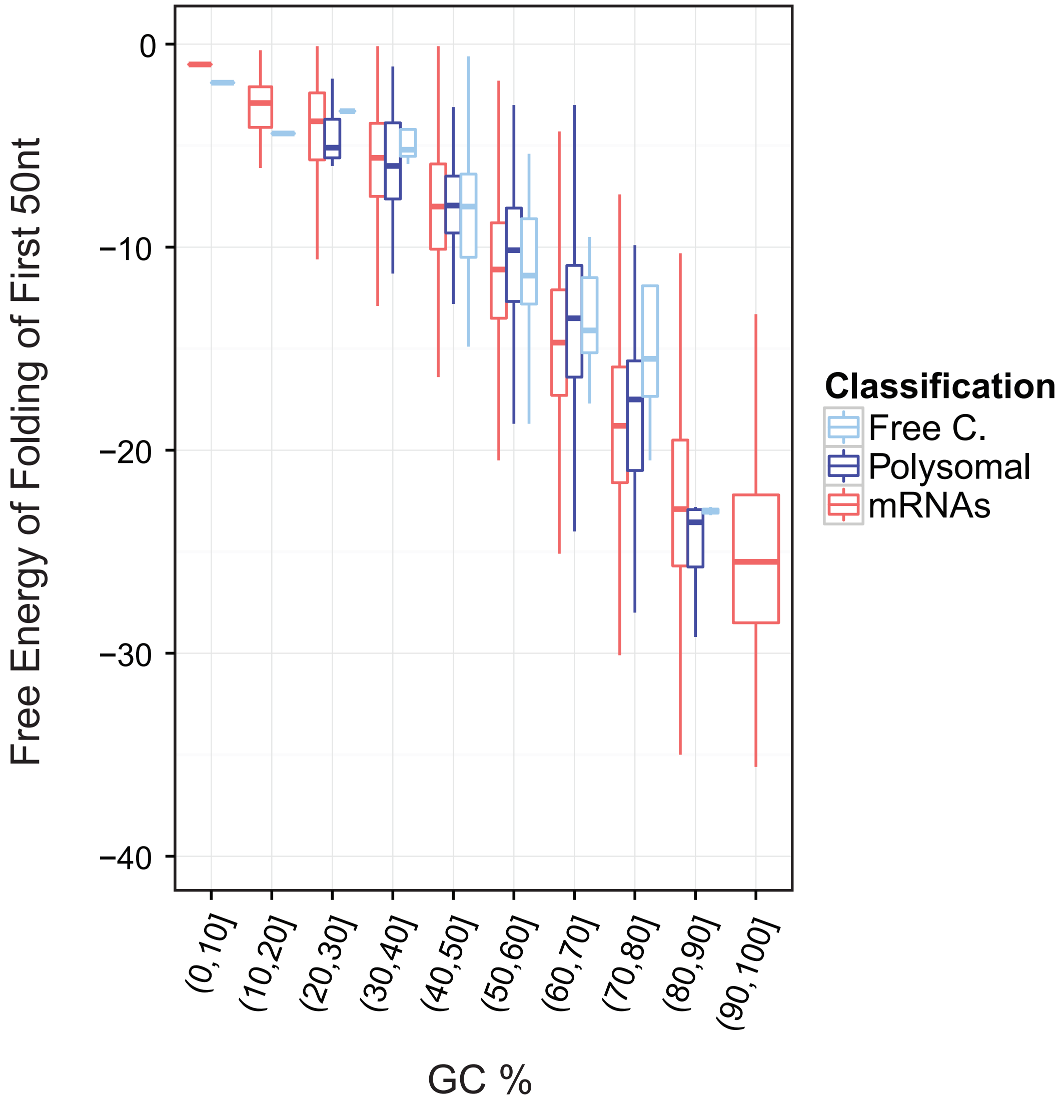

Figure S15

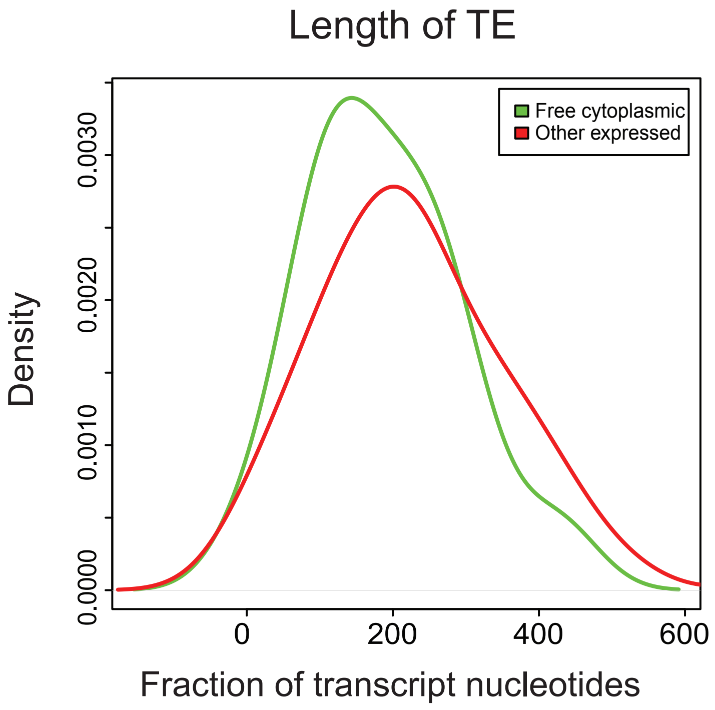

Length of TE
Free cytoplasmic
Other expressed
Density
0.0000
0.0010
0.0020
0.0030
0
200
400
600
Fraction of transcript nucleotides

Figure S16

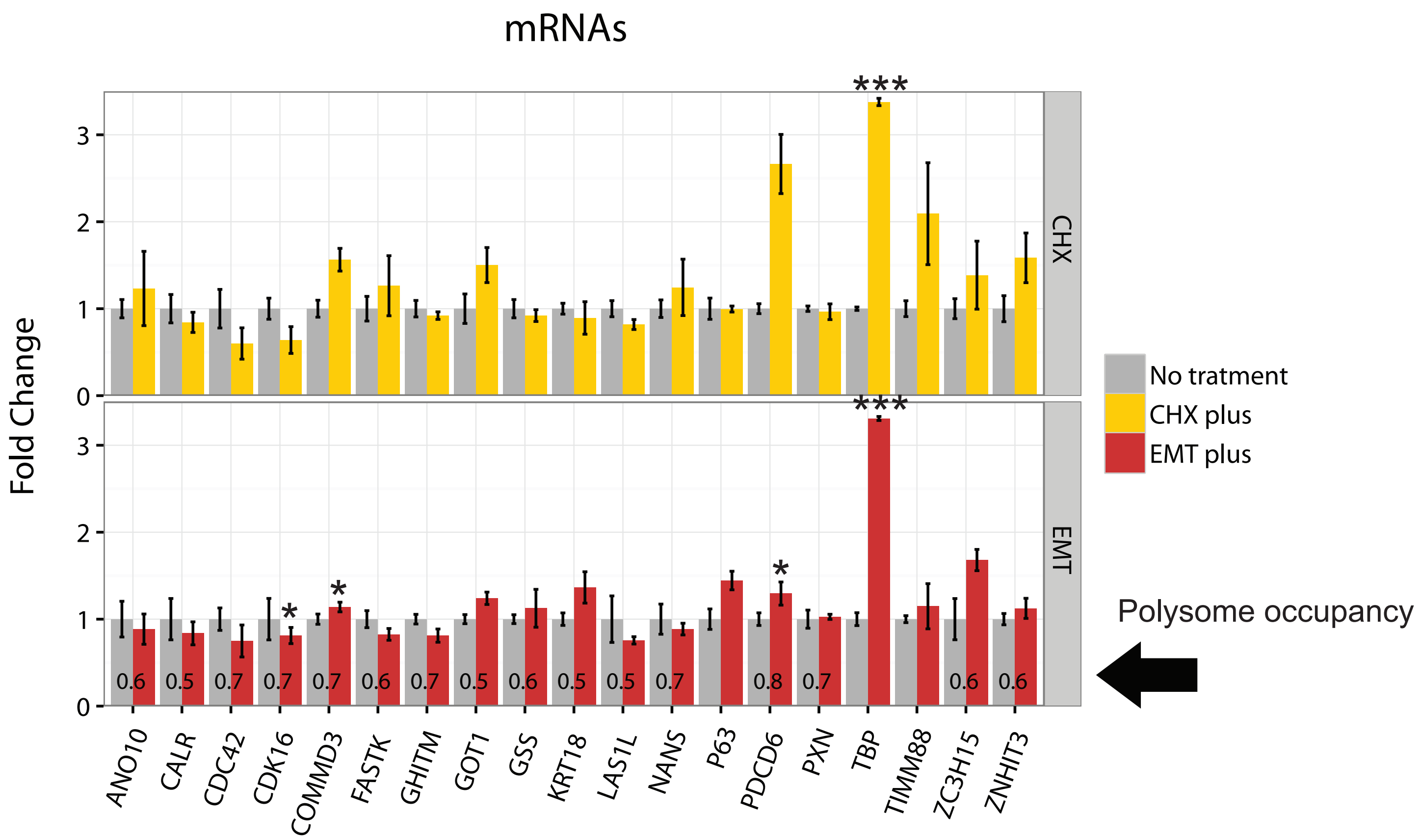

mRNAs
***
***
*
*
*
Fold Change
CHX
EMT
No tratment
CHX plus
EMT plus
Polysome occupancy
0.6
0.5
0.7
0.7
0.7
0.6
0.7
0.5
0.6
0.5
0.5
0.7
0.8
0.7
0.6
0.6
ANO10
CALR
CDC42
CDK16
COMMD3
FASTK
GHITM
GOT1
GSS
KRT18
LAS1L
NANS
P63
PDCD6
PXN
TBP
TIMM88
ZC3H15
ZNHIT3

Figure S17

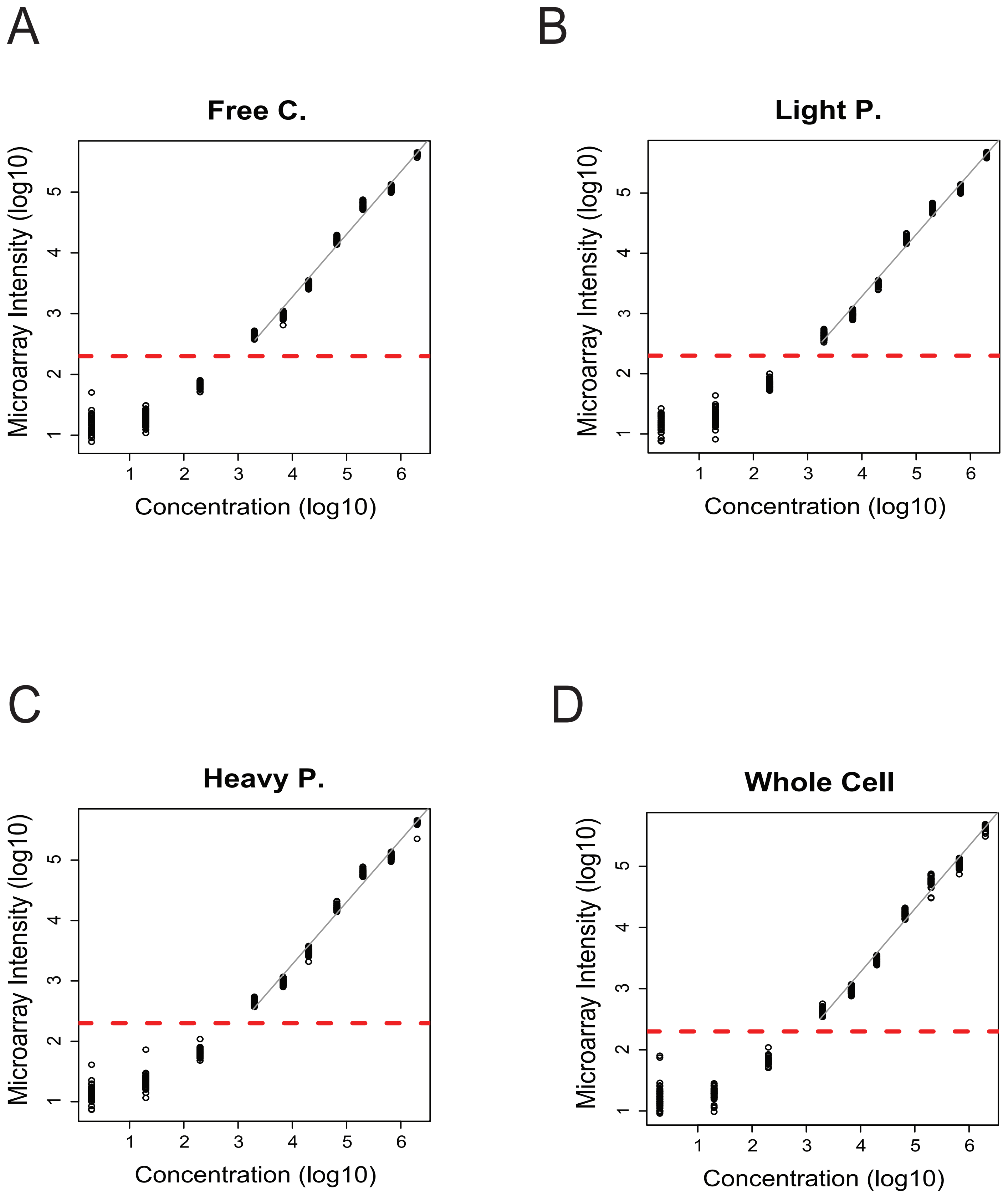

A
Free C.
Microarray Intensity (log10)
Concentration (log10)
B
Light P.
Microarray Intensity (log10)
Concentration (log10)
C
Heavy P.
Microarray Intensity (log10)
Concentration (log10)
D
Whole Cell
Microarray Intensity (log10)
Concentration (log10)
