# Supplementary material for: Cytoplasmic long noncoding RNAs are frequently bound to and degraded at ribosomes in human cells
Source: RNA. 2016 Jun;22(6):867–82. doi: 10.1261/rna.053561.115 (PMC4878613; doi:10.1261/rna.053561.115)
Supplement: Supplemental Material [file supp_053561.115_Supp_Legends.doc]

**SUPPLEMENTARY DATA FILES**

**Table S1: Gencode v7 lncRNA classification.** Rows represent lncRNA transcript from Gencode v7. Genomic coordinates refer to human genome version (hg19). Columns:

TransID: ENST ID for transcripts.

GeneID: ENSG ID for the corresponding gene.

Chr: Chromosome.

Trans_Start: Transcript start position.

Trans_End: Transcript end position.

Strand

Cellular_Localization: Classification of the transcripts into 5 different categories: 1: Present in cytoplasm (from polysome profiling experiment, K562 cell line); 2: Present in nucleus (from ENCODE nucleus RNAseq data, K562 cell line), but not in cytoplasm (polysome profiling experiment); 3: Not present in cytoplasm (polysome profiling experiment) nor in nucleus (ENCODE nucleus RNAseq data, K562 cell line); 4: Transcripts classified as potential protein coding transcripts; 5: Discarded transcripts. See Materials and Methods for details.

FreeC_Conc: Log10 concentration value for free C. condition (NA if not present in this condition).

LightP_Conc: Log10 concentration value for Light P. condition (NA if not present in this condition).

HeavyP_Conc: Log10 concentration value for Heavy P. condition (NA if not present in this condition).

Ribosomal_Classification: Classification for transcripts present in the cytoplasm: 1: free C.; 2: Light P.; 3: Heavy P.

CPAT: CPAT score.

PhyloCSF: PhyloCSF score.

CPC: CPC score.

MS: information about presence of peptides in Mass Spectrometry analysis for this transcripts: 0: No peptide associated; 1: Peptide associated.

**Figure S1: Consistency of polysome profiling measurements from weak and strong lysis buffer conditions, for mRNAs.** Comparison of polysome profiling results of microarray detected protein coding genes with high- and low-stringency cell lysis buffers. The figures show, for every transcript, the measured microarray value in high- (x axis) and low-stringency buffer samples (y axis).

**Figure S2: Consistency of polysome profiling measurements from weak and strong lysis buffer conditions, for lncRNAs.** Comparison of polysome profiling results of microarray detected lncRNAs with high- and low-stringency cell lysis buffers. The figures show, for every transcript, the measured microarray value in high- (x axis) and low-stringency buffer samples (y axis).

**Figure S3: Consistency in transcript classification between weak and strong lysis experiments.** The same classification analysis was performed on both experiments, and the results were compared. Barplots represent numbers of lncRNA transcripts and protein coding genes (mRNA). Different colours represent groups of transcripts classed by their change on classification between the two lysis conditions. F refers to Free Cytoplasmic, L to Light Polysomal and H to Heavy Polysomal classifications. “Undetected” are those transcripts or genes that from one experiment to the other are no longer detected.

**Figure S4: Polysome occupancy of mRNAs correlates positively with translation index.** Barplot shows Pearson Correlation coefficients obtained from testing the correlation between fraction occupancy from a given fraction and translation index of ribosome-profiled mRNAs classified to be maximally detected in that fraction (* P < 0.05; ** P < 0.01). Translation index is defined as the peptide count (assayed by mass spectrometry) normalised to RNA levels (assayed by RNAseq), both in K562 cells.

**Figure S5: Potential protein coding transcripts across cytoplasmic and ribosomal fractions.** Heatmap showing potential protein coding transcripts concentration (log10) measured for each RNA cytoplasmic fraction. Only transcripts detected at least in one cytoplasmic fraction are shown.

**Supplementary Figure S6: Independent evidence of cytoplasmic expression and association to ribosomes for XIST and MALAT1 known lncRNAs.**

Genomic map from UCSC Genome Browser displaying subcellular compartment expression from ENCODE and ribosome footprinting signal from GWIPS-viz database showing lncRNAs, XIST and MALAT1, and an example of a well-known coding, cytoplasmic and translated gene, GAPDH. Probes from the custom microarray design used in this study are also displayed.

**Figure S7: XIST ribosomal interaction validation through puromycin treatment.** Two replicate experiments were carried out with control K562 (red) and cells treated with puromycin (blue), for three distinct RNA fractions: (from left to right) free cytoplasmic, light polysomal, heavy polysomal. XIST RNA levels are normalized to absolute levels of an RNA spiked into equal volumes of RNA sample.

**Figure S8: Expression of lncRNA across 16 Human Body Map tissues.** (A) Median of transcripts expression in Human Body Map tissues. For protein coding transcripts data is only shown for those transcripts detected in K562. (B) Heatmaps show log10 RPKM expression measured for each Human Body Map tissue sample for all free cytoplasmic transcripts and polysomal transcripts separately.

**Figure S9: Association of ORF length with polysome density for mRNAs.** (A) Shown are histograms for nucleotide length of longest predicted ORF in coding transcripts. Data are shown for mRNAs included in microarray design and classified by ribosomal occupancy. (B) Same as A, but shown as cumulative plot.

**Figure S10: Association of ORF length with polysome density for lncRNA transcripts.** As for Figure S9, but considering lncRNAs.

**Figure S11: Association of ORF coverage with polysome density for lncRNA transcripts.** Shown are histograms for % coverage of transcript length by their longest ORF.

**Figure S12: GC content of free cytoplasmic and polysomal lncRNA transcripts.**

**Figure S13: Splicing efficiency of lncRNAs.** Using ENCODE data, we calculated RPKM values separately for the exons and introns of all lncRNAs. Shown are the log10 ratios of exon/intron values for all sets of transcripts. No statistically significant differences were found between free cytoplasmic lncRNAs and polysomal lncRNAs using either the Kolmogorov-Smirnov or Wilcoxon tests.

**Figure S14: Comparison of 5’ RNA folding energy.** Using the Vienna RNAfold programme with default settings, we estimated the free energy of folding of the first 50nt of lncRNA and mRNA. (A) Cumulative frequency comparing free energy of folding of each group. While mRNA have more stable folding on average than expressed lncRNA (P=2.2e-16, Wilcoxon test), we could find no statistically significant difference between polysomal transcripts and free cytoplasmic lncRNAs. (B) Boxplot showing free energy of folding of mRNAs, polysome-associated and free cytoplasmic lncRNAs divided into bins according to their GC content. This analysis is designed to account for the differences in average GC content between mRNA and lncRNA. When analysing in this way, the dissimilarity between mRNAs and lncRNAs is lost.

**Figure S15: ERVL-MaLR insertion length distributions.**

**Figure S16: Changes in mRNA stability in response to drug-induced ribosome stalling.**

K562 cells were treated with cycloheximide (CHX) or emetine (EMT) as described in the Materials and Methods. Results are show for coding genes. Statistical significance was calculated by one-sided t-test (* P<0.05, ** P<0.01, *** P<0.001).

**Figure S17: Spike-in linear models for normalisation of microarray data.** For each spike-in sample we plotted microarray intensity signal of its probes after normalization (y axis) versus their known initial concentration (x axis). Horizontal slashed red line shows the detection cutoff of each sample, defined at the lowest spike in with linear detection characteristics. Only spike-in probes above the cutoff were taken into account to compute the linear model used to predict concentration values from microarray intensity values.
